# The Clinical Use of Platelet-Rich Plasma in Knee Disorders and Surgery—A Systematic Review and Meta-Analysis

**DOI:** 10.3390/life10060094

**Published:** 2020-06-25

**Authors:** Ewa Trams, Krzysztof Kulinski, Katarzyna Kozar-Kaminska, Stanislaw Pomianowski, Rafal Kaminski

**Affiliations:** 1 Department of Orthopaedics and Trauma Surgery, Centre of Postgraduate Medical Education, Professor A. Gruca Teaching Hospital, Konarskiego 13, 05-400 Otwock, Poland; ewa.trams@gmail.com (E.T.); k.kulinski@o2.pl (K.K.); spom@spskgruca.pl (S.P.); 2 Department of Medical Biology, Stefan Cardinal Wyszynski National Institute of Cardiology, ul. Alpejska 42, 04-628 Warsaw, Poland; k.kozar@ikard.pl

**Keywords:** PRP, platelet-rich plasma, meniscus, anterior cruciate ligament (ACL), osteoarthritis, tendinopathy, arthroscopy, knee lesion, total knee arthroplasty, osteoarthritis (OA), meniscal repair

## Abstract

In recent years, the interest in biological treatment of knee lesions has increased, especially the application of platelet-rich plasma is of particular note. The number of articles evaluating platelet-rich plasma (PRP) efficacy in the recovery of knee disorders and during knee surgery has exponentially increased over the last decade. A systematic review with meta-analyses was performed by assessing selected studies of local PRP injections to the knee joint. The study was completed in accordance with 2009 Preferred Reporting Items for Systematic Reviews and Meta-Analyses (PRISMA) statement. A multistep search of PubMed, Embase, Cochrane Database of Systematic Reviews, and Clinicaltrials.gov was performed to identify studies on knee surgery and knee lesion treatment with PRP. Of the 4004 articles initially identified, 357 articles focusing on knee lesions were selected and, consequently, only 83 clinical trials were analyzed using the revised Cochrane risk-of-bias tool to evaluate risk. In total, seven areas of meta-analysis reported a positive effect of PRP. Among them, 10 sub-analyses demonstrated significant differences in favor of PRP when compared to the control groups (*p* < 0.05). This study showed the positive effects of PRP, both on the recovery of knee disorders and during knee surgery; however further prospective and randomized studies with a higher number of subjects and with lower biases are needed.

## 1. Introduction

Knee disorders are among the most frequent disorders treated by orthopedic surgeons. Traumatic knee injuries, as well as knee degeneration, require special attention and appropriate treatment. The first line of treatment is usually conservative and includes physical therapy, rehabilitation, braces or non-steroid inflammatory drugs. Recently, orthobiologics—naturally occurring substances in the body—were introduced to clinics [1,2]. One type of orthobiologic substance, platelet-rich plasma (PRP) shows promising results for minimally invasive treatment of knee lesions through enhanced healing potential of damaged cartilage, tendons, and ligaments [1]. PRP, also known as platelet-rich fibrin (PRF), platelet concentrate or platelet-rich growth factors (PRGFs) is a concentration of platelets derived from the patient’s whole blood, which has to be centrifuged to obtain a ready-to-use product [2,3]. The mechanism of action relies on releasing cytokines and growth factors from alpha granules such as interleukin 1β, interleukin 8, tumor necrosis factor (TNF-α), platelet derived growth factor (PDGF), platelet derived endothelial growth factor (PDEGF), transforming growth factor β1 (TGF-β1), insulin-like growth factor 1 (IGF-1), fibroblast growth factor 2 (FGF-2), hepatocyte growth factor (HGF), and vascular endothelial growth factor A (VEGF-A). These enhance healing by stimulating cell proliferation, migration, and differentiation, alongside interaction with the immune system, inflammation, and angiogenesis [1,2,3,4]. Possible indications for PRP application in knee disorders and knee surgery are cartilage degeneration in osteoarthritis and soft tissue injuries in sports medicine. Well documented clinical trials are related to patients with degenerative meniscus lesions, patellar tendinopathy, graft remodeling in anterior cruciate ligament (ACL) reconstruction, hamstring tendinopathy, and medial collateral ligament (MCL) injuries [1,5]. There is also some evidence for pain reduction after total knee arthroplasty (TKA) and bone remodeling after osteotomies. Several systematic reviews and meta-analyses have been published, although with contradictory results; therefore, we aimed to elucidate these controversial issues and performed a systematic review and meta-analysis on the efficacy of PRP use in disorders around the knee.

## 2. Results

### 2.1. Literature Search

**Figure 1:**
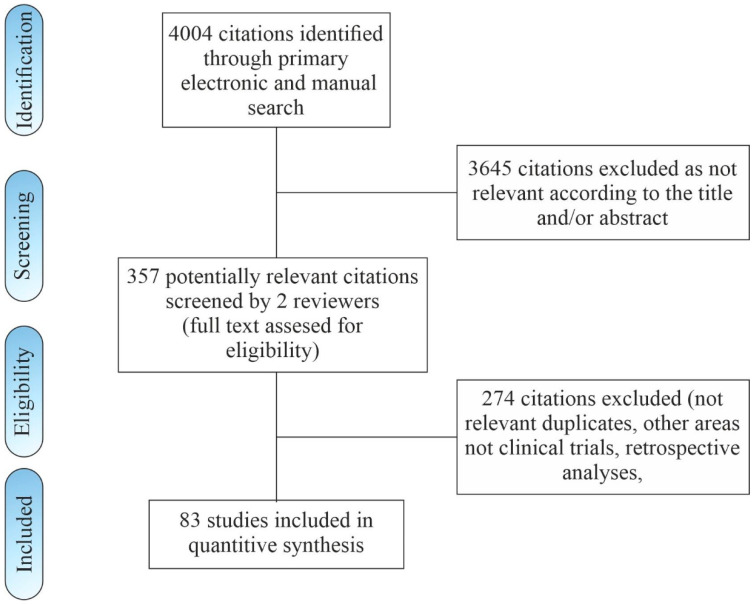
Flow chart of study inclusion.

A literature search through electronic databases identified a total of 4002 records according to the selected search algorithm and two additional studies were included through reference list evaluation. A total of 3645 citations was excluded as irrelevant according to title and/or abstract. The abstracts of 357 remaining articles were assessed for eligibility. From these, 274 were excluded. The remaining 83 clinical studies published between 2005 and 2020 with 5323 patients were included in this review. The literature search flowchart is shown in Figure 1.

### 2.2. Study Characteristics

A total of 83 randomized controlled trials (RCTs) and seven non-RCTs was included in our study. The characteristics of the selected articles are summarized in Table 1, Table 2, Table 3, Table 4, Table 5, Table 6, Table 7, Table 8 and Table 9. All of the selected studies were included into a systematic review. Mean follow-up period was 12 months (ranging from 10 days to 3 years) and the mean number of patients included was 62 (ranging from 20 to 315). 

One injection of platelet-rich plasma was performed in 55 studies, two injections in 14 studies, three injections in 21 studies and four injections in two studies. Platelet concentration was provided in 48 articles, 33 studies used leukocyte-rich PRP, 25 studies used leukocyte-poor PRP, and in 25 studies no information was provided.

In addition, 41 studies compared the application of PRP versus other treatments (25 versus hyaluronic acid (HA), 4 versus corticosteroids, 4 versus microfractures, 10 versus other substances), 42 studies compared the use of PRP versus placebo (12 versus saline and 30 versus no injection), and 7 studies compared single injection of PRP versus multiple injections. Primary outcomes included pain measurement (visual analog scale (VAS)) in 48 studies and functional outcomes in 73 studies: International Knee Documentation Committee (IKDC), 24 studies; Western Ontario and McMaster Universities Osteoarthritis Index (WOMAC), 32; Victorian Institute of Sport Assessment for patella tendonitis (VISA-P), 5; 36-Item Short Form Survey (SF-36), 7; Knee injury and Osteoarthritis Outcome Score (KOOS), 12; The Lysholm Knee Scoring Scale, 14; Teger Activity Score, 10; Lequesne score, 6; and others (meniscal repair failure, 6; time for return to sport (RTS), 4; re-injury, 3; knee stability, 6; graft integration, 5; tunnel widening, 4; hemoglobin drop, 6; range of movement (ROM), 9). Radiographic outcomes were presented in 15 studies (computed tomography, X-ray, magnetic resonance imaging, ultrasonography). 

A total of 75 studies was included into quantitative synthesis: VAS was analyzed in 5 subgroups, IKDC, 5; WOMAC, 3; Tegner, 1; KOOS (activities of daily living (ADL), 1; pain, 1; quality of life (QoL), 1; sport, 1; symptoms, 1); VISA-P, 1; SF-36, 1; graft integration, 1; tunnel widening, 1; re-injury rate, 1; RTS, 1; repair failure, 1; blood loss, 1; KT-1000 (knee arthrometer), 1; adverse events, 1.

### 2.3. Patellar Tendinitis (PT)

Four studies reported data from 137 patients. Inclusion criteria required randomization, control groups, use of VAS for pain as well as VISA-P with a minimum of 6 months follow-up. We included RCTs comparing the use of PRP in patellar tendinopathy versus saline, dry needling (DN) or extracorporeal shockwave therapy (ESWT) (Table 1).

**Figure 2:**
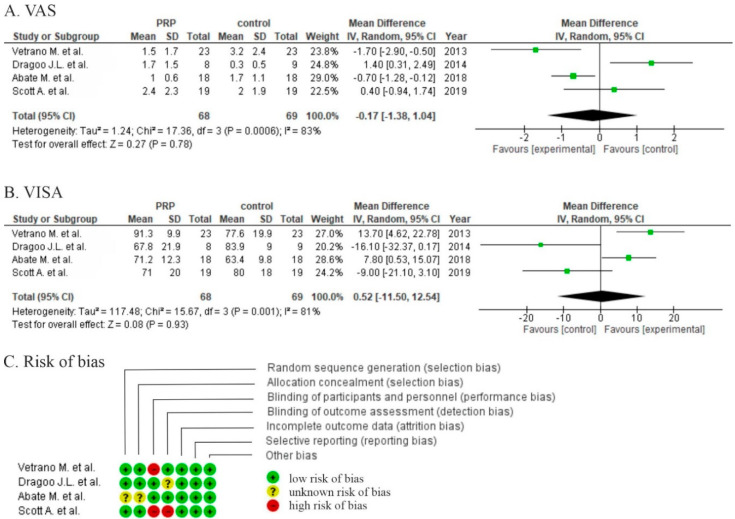
Forest plot for (**A**) visual analog scale (VAS) scores and (**B**) Victorian Institute of Sport Assessment (VISA) (CI: confidence interval; IV: inverse variance; SD: standard deviation.). (**C**) Risk of bias analysis.

Two studies showed non-significant differences in favor of PRP (*p* > 0.05) in VAS comparing PRP with saline injection after 1 year [6] or DN after 6 months [7]. Two studies also reported pain scales (VAS) with significant differences at, respectively, 1 year compared to ESWT (*p* = 0.009) [8] and 6 months compared to high volume image guided injections (HVIGIs) [9]. The pooled estimate for these 4 studies demonstrated non-significant differences in favor of PRP (*p* = 0.80) (Figure 2A).

The same authors measured the severity of jumper’s knee via VISA-P score. Two studies [6,7] proved no differences in symptom severity after 6 months and 1 year with statistical significance greater than 0.05. Another study showed significant differences between groups of PRP injection and ESWT (*p* = 0.026) after 1 year [8] and significant differences as compared to HVIGI (*p* = 0.03) [9]. Pooled data estimated for these studies demonstrated non-significant differences in favor of PRP (*p* = 0.93) (Figure 2B).

Functional outcomes with Tegner, Lysholm, and SF-12 scores were analyzed in one study. Dry needling showed significant improvement at >26 weeks when compared to PRP group (*p* = 0.006) [7]. In another study, a modified Blazina scale showed significant improvement at 12 months in favor of the PRP group (*p* = 0.015) [8].

Two studies were at high risk of bias for one or more domains [6,8], and two studies were at an unclear risk of bias for one or more domains (Figure 2C). Moderate risk of performance bias was identified in two studies [6,8]. Similarly, two were at risk of detection bias [6,7]. No data concerning the generation of random sequence and allocation were provided thus increasing risk of selection bias [9]. 

### 2.4. Muscle Injuries around the Knee

Four studies including 224 patients measured time for return to sport after a muscle injury (hamstring, quadriceps, gastrocnemius). In all reported studies PRP was delivered intralesionally. Two studies performed the injection under the guidance of ultrasound [10,11] and the other two used magnetic resonance imaging (MRI) prior to the injection to detect the damaged area [12,13]. Three studies reported re-injury incidences, and only two provided patient reported outcome measures (pain). Each study compared rehabilitation programs with/without PRP injection. All reported shorter time for return to sport in favor of PRP in comparison to control groups (Table 2). One study included only professional athletes [12] and three studies recruited both competitive and recreational athletes [10,11,13].

**Figure 3:**
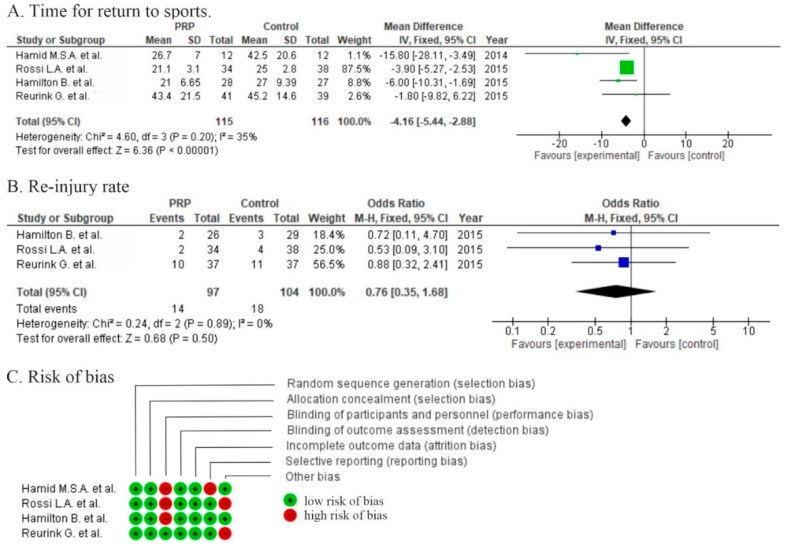
Forest plot for (**A**) time for return to sport (RTS) and (**B**) re-injury rate (CI: confidence interval; IV: inverse variance; SD: standard deviation). (**C**) Risk of bias analysis.

The mean time for return to sport ranged from 21 to 43 days in the PRP group and from 25 to 45 days in the control groups. Two studies [10,11] showed significant differences in RTS (*p* = 0.001; *p* = 0.02) and two studies [12,13] showed shorter RTS, but no significant differences between PRP and control groups (*p* > 0.05). The pooled estimate for these 4 studies demonstrated significant differences in favor of PRP (*p* ≤ 0.00001) with a mean difference of −4.16 (−5.44, −2.88) (Figure 3A). Due to the high heterogeneity of patient recruitment and only small differences in the time to return to sport, an analysis of cost-effectiveness should be accomplished to evaluate whether the results are worth the cost. 

The re-injury rate ranged from 6% to 27% in the PRP group and from 10% to 31% in the control groups. Three studies [10,12,13] reported lower re-injury rate in favor of the PRP group but with non-significant differences (*p* = 0.47) (Figure 3B).

Two studies [10,11] showed significantly lower pain severity (beta regression coefficient = −0.272, 95% confidence interval (CI) (−0.5, −0.045), *p* = 0.019 during motion and −0.390, 95% CI (−0.67, −0.11), *p* = 0.007, respectively) but non-significant differences in pain intensity (*p* = 0.157) [13].

Two studies were at high risk of bias for two domains, and two studies were at high risk for one domain (Figure 3C). Moderate risk of performance bias was identified in three studies [10,11,12]. One was at risk of reporting bias [11]. Discrepancies between the number of patients undergoing final follow up in Preferred Reporting Items for Systematic Reviews and Meta-Analyses (PRISMA) chart versus manuscript was detected in two studies [10,13]. 

### 2.5. High Tibial Osteotomy (HTO)

Two RCTs including 80 patients evaluated the intraoperative use of PRP as an adjunct to HTO with or without the addition of other myeloid stromal cells [14,15] (Table 3).

Koh et al. injected PRP into the medial joint space under arthroscopic visualization and afterwards performed HTO. This study showed significant differences in KOOS and VAS in favor of PRP with the addition of Mesenchymal Stem Cells (MSC) in a 2-year follow up (*p* < 0.05). Second-look arthroscopy during plate removal reported a significant difference between the groups with respect to cartilage healing again in the PRP + MSC groups (*p* = 0.023) [14]. 

Dallari et al. added lyophilized bone chips with platelet gel and with/without bone marrow (BM) to the osteotomy hole. This study showed better osseointegration in X-ray analysis after 1-year follow up and histologically more active osteogenic processes in favor of PRP+/−BM groups (*p* < 0.05) [15]. 

Both studies were at high risk of bias for one domain (performance bias). 

### 2.6. Total Knee Arthroplasty (TKA)

Six RCT studies including 621 patients evaluated the use of PRP as an adjunct to TKA (Table 4). All studies applied PRP intraoperatively: three sprayed platelet gel onto the exposed surface of the wound [16,17,18] and the other three injected PRP into the joint [19,20,21]. The aim of all these studies was to assess potential blood loss during the procedure after TKA.

**Figure 4:**
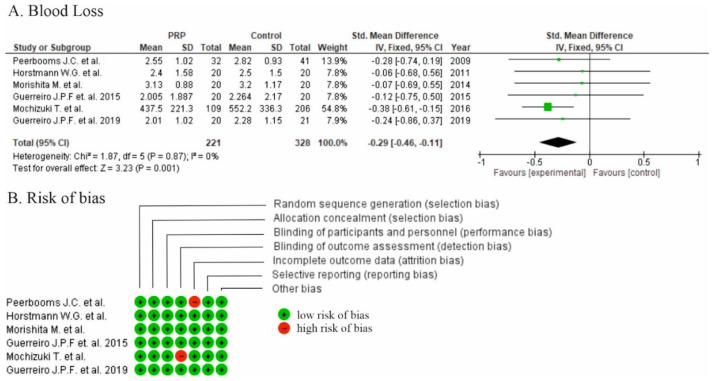
(**A**) Forest plot of time for blood loss analysis after total knee arthroplasty (TKA) (CI: confidence interval; IV: inverse variance; SD: standard deviation). (**B**) Risk of bias analysis.

Lower hemoglobin drop was reported in all six studies [16,17,18,19,20,21] with significant differences and two studies reported lower calculated blood loss in the PRP group (*p* > 0.05; *p* < 0.001) [17,21]. There were significant differences in favor of PRP in comparison to the control groups for the overall effect on blood parameters (standardized mean difference −0.29, 95% CI (−0.46, −0.11), *p* = 0.001) for the pooled estimates for all six studies (Figure 4A). 

Four studies reported better pain control in the PRP group (VAS) for a short time period after surgery [16,18,19,20]. No effect was observed in long-term follow-up. 

Functional outcome was measured using the WOMAC [18,19,20] score in three studies and Knee Society Score (KSS) and KOOS in another study [17], but with non-significant differences between the groups. Range of motion was measured in all studies with non-significant differences between the groups [16,17,18,19,20,21]. Thromboembolism was absent in all studies. However, Morishita et al. reported one patient requiring a secondary skin suture in the PRP group [17]. Peerbooms et al. and Guereirro et al. reported superficial wound infection in one and two patients, respectively, all treated successfully with antibiotics [18,19]. A subsequent study performed by Guerreiro reported two cases of deep infection treated by debridement and TKA review [20].

Two studies were at high risk of bias for one domain (Figure 4B). Low risk of performance bias was identified in four studies [16,17,19,20]. A moderate risk of performance bias was identified in two studies [18,21] (Figure 4B).

### 2.7. Arthroscopy

Four RCTs [22,23,24,25,26,27] and three prospective cohort [26,27,28] studies, including 199 patients, evaluated the use of PRP as an adjunct to knee arthroscopy treatment for cartilage and/or meniscal pathology: two studies included patients with osteoarthritis (OA) changes according to the Kellgren and Lawrence (KL) classification system with concomitant meniscal lesions [22,23]; two studies included patients with cartilage lesions of grade III–IV according to the Outerbridge classification system and early OA stages I–II according to the KL classification system [24,27]; one study included patients with chondral defects of medial femoral condyle grade II–III according to the Outerbridge classification system [25]; and one study included patients with OA stage II–III according to the KL classification system [26]. In five studies [23,24,26,27,28] PRP was used intraoperatively, and in another two studies PRP was used after surgery [22,25]. Kim et al. was excluded from the meta-analysis. The study analyzed PRP effectiveness when applied as an adjuvant to injection of MSC versus surgical implantation of MSCs [28].

**Figure 5:**
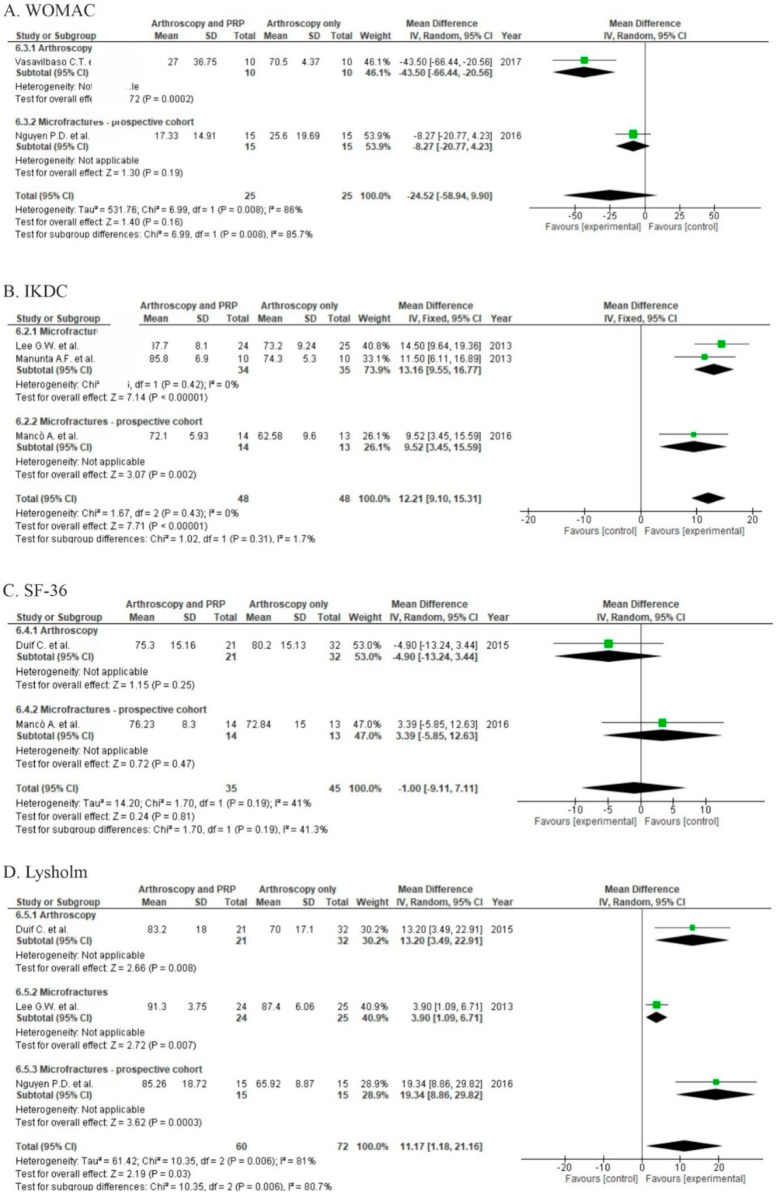
Forest plot for (**A**) Western Ontario and McMaster Universities Osteoarthritis Index (WOMAC), (**B**) International Knee Documentation Committee (IKDC), (**C**) 36-Item Short Form Survey (SF-36), and (**D**) Lysholm scores (**D**) (CI: confidence interval; IV: inverse variance; SD: standard deviation).

Two studies reported functional outcome in the WOMAC score [22,26]. One of them (RCT) showed significant differences in WOMAC scores (*p* = 0.0002) when comparing PRP to a control group at 18 months and reported hyaluronic acid injections to be more effective than PRP [22]. The pooled estimate for these two studies showed significant differences in favor of the PRP group (*p* = 0.0040, Figure 5A). Four studies [24,25,27,28] reporting outcomes measured in IKDC (Figure 5B), showed significant differences in favor of PRP (*p* < 0.00001). In subgroup analysis, two RCTs presented significant differences in favor of PRP when applied with microfractures [24,25]. Additionally, one prospective cohort trial also showed significant differences in favor of PRP [27]. Another two studies [23,27] showed better outcomes in patient self-assessment SF-36 scale, one of them in favor of the control [27] and the other in favor of the PRP group [23]; but differences were not significant (*p* = 0.81, Figure 5C). Functional outcome was also measured by the Lysholm score by three studies [23,24,26] (Figure 5D), showing non-significant differences in favor of PRP (*p* = 0.03).

**Figure 6:**
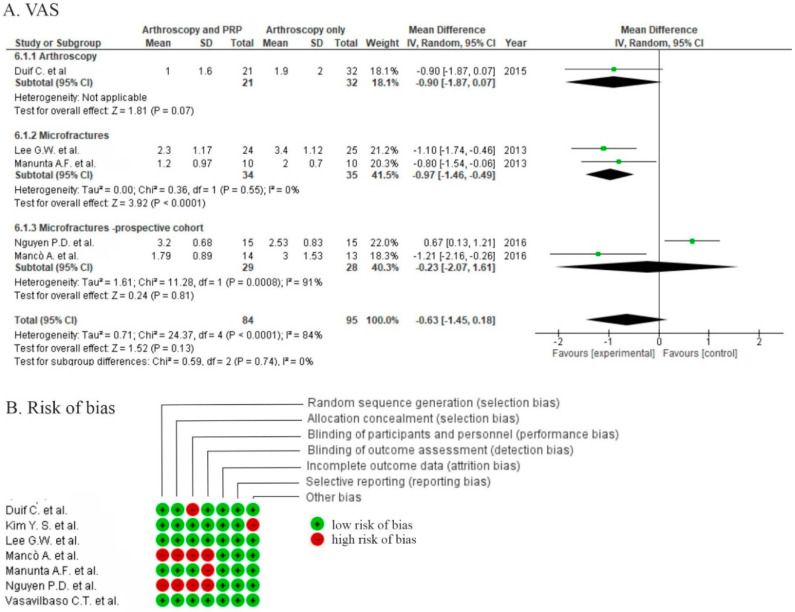
(**A**) Forest plot pain intensity for VAS (CI: confidence interval; IV: inverse variance; SD: standard deviation). (**B**) Risk of bias analysis.

Three randomized studies [23,24,25] and two prospective cohort studies [26,27] used VAS to assess pain level. Two studies [24,25] with the addition of PRP to arthroscopic microfractures showed significant differences in pain severity in favor of PRP (*p* < 0.0001); although two cohort studies report non-significant differences in favor of PRP (*p* = 0.81). Arthroscopy without microfractures showed lower pain levels when complemented with PRP, but the differences were not significant (*p* = 0.07) [23]. The pooled estimate for these five studies demonstrated non-significant differences in favor of the PRP group (*p* = 0.13) (Figure 6A). Due to the large variety of patient recruitment regimens, any conclusions should be stated carefully. However, all subgroups showed positive effects of PRP during synthesis. There is a need for more RCTs to allow for definitive conclusions with low heterogeneity.

Two studies were at high risk of bias for four domains [26,27] and three studies were at high risk of bias for one domain [23,25,28]. High risk of performance bias was identified in two studies [26,27], moderate risk of performance bias was identified in three studies [23,25,28], and a low risk of performance bias was identified in two studies [22,24] (Figure 6B).

### 2.8. Anterior Cruciate Ligament Reconstruction (ACL)

Sixteen RCTs, including 740 patients, evaluated the use of PRP as an adjunct to ACL reconstruction with patellar ligament (Bone-Patella Tendon-Bone-BPTB) autograft [29,30,31,32,33,34,35] or hamstrings graft [33,36,37,38,39,40,41,42,43,44] (Table 6). 

**Figure 7:**
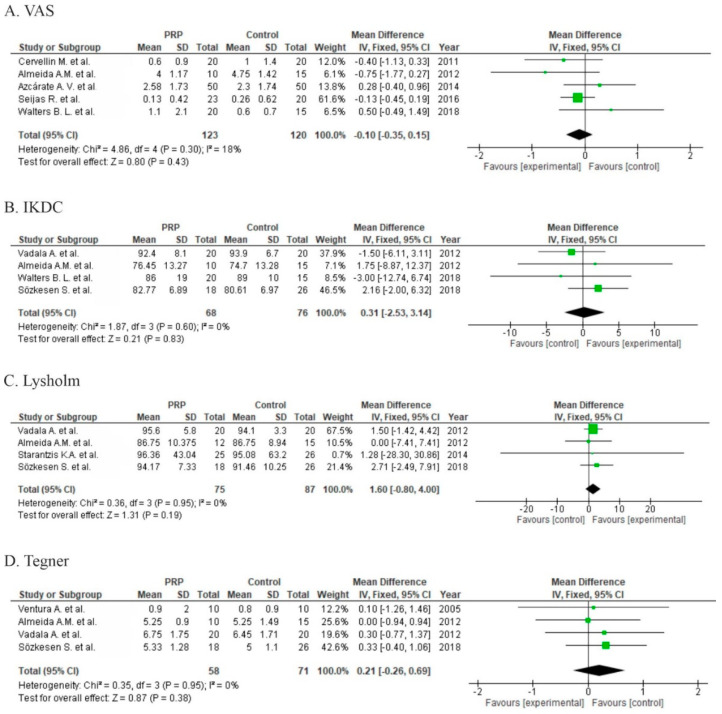
Forest plot for (**A**) VAS, (**B**) IKDC, (**C**) Lysholm score, and (**D**) Tegner scores (CI: confidence interval; IV: inverse variance; SD: standard deviation).

Five studies reported pain assessment with the VAS [29,30,31,32,34]. The overall effect showed no significant differences with respect to pain (*p* = 0.43); however, two studies showed significant differences in short-term follow-up in favor of the PRP group (2–6 months) [31,32] (Figure 7A).

Seven studies reported functional results via IKDC scores [30,32,34,38,39,43,44], but only four of them provided data allowing for synthesis [30,32,43,44] (Figure 7B) and no significant differences (*p* = 0.83) were detected. A further four studies provided only categorical output data (excellent, good, regular, poor) with non-significant odds ratio (1.39 (0.27, 7.21), *p* = 0.7). Functional outcome was measured by the Lysholm score in four studies and provided insignificant results (*p* = 0.19, Figure 7C). Five studies used the Tegner scale for activity assessment [38,42,43,44,45]. Pooled estimates for these studies showed no significant differences (*p* = 0.38) in favor of the control (Figure 7D). Three studies showed no significant differences [38,42,43] in functional outcomes, one study did not report functional outcome results [42], and one study reported worse outcomes in both groups when compared to baseline [32]. 

**Figure 8:**
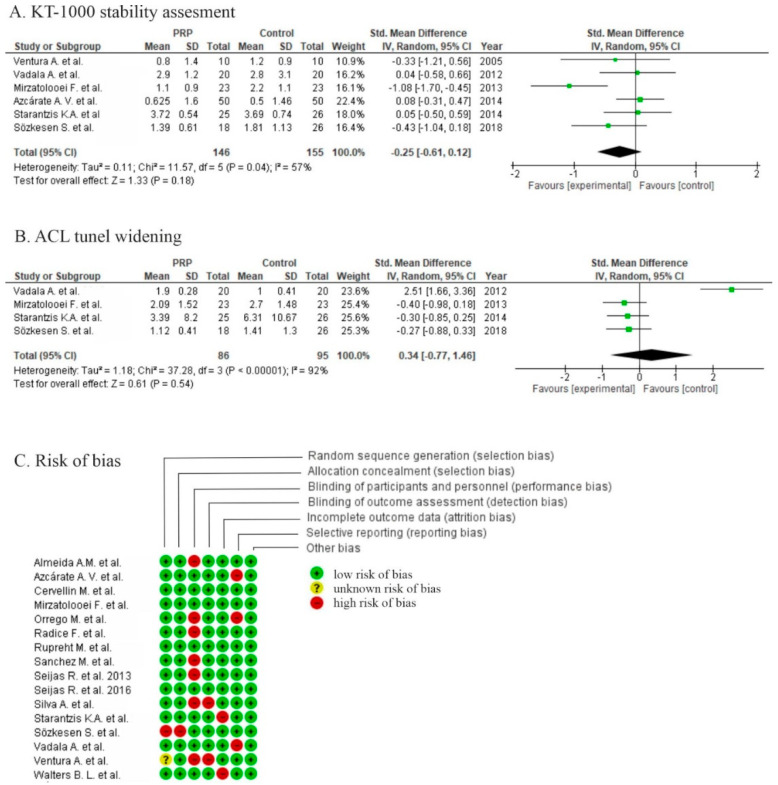
Forest plot for (**A**) KT-1000 (knee arthrometer) stability assessment and (**B**) tunnel widening (CI: confidence interval; IV: inverse variance; SD: standard deviation). (**C**) Risk of bias analysis.

Six studies reported the outcomes of anterior tibial translation assessments [34,38,41,42,43,44] with no significant differences between groups (*p* = 0.18) in meta-analysis. Only one study showed a significant difference in favor of PRP using KT-1000 (Figure 8) [41].

Five studies reported the outcome of tunnel widening after graft fixation, two of them used computer tomography (CT) [43,44] and three used MRI [39,41,42] to evaluate tunnel enlargement. The pooled estimates for four studies included in meta-analyses showed non-significant differences in favor of the control (*p* = 0.54) (Figure 8).

Eight studies assessed the outcomes of ACL graft integration in the femoral or tibial tunnel. Six of them evaluated signal intensity of the graft on MRI [33,34,35,36,37,39], one reported significant difference in ACL density measured on CT (*p* < 0,01) [38], and one explored better remodeling using histologic parameters (*p* = 0.024) [40]. Three studies [33,39,40] reported faster graft remodeling (*p* < 0.001; *p* = 0,036; *p* = 0.024), and the remaining four [34,35,36,37] showed no significant differences during the final follow-up. We included in the meta-analysis four studies [35,36,39,40] and the pooled estimates for these studies showed non-significant differences in favor of PRP (*p* = 0.06).

Three studies were at high risk of bias for two domains [36,39,43], eight studies were at high risk of bias for one domain [30,32,33,34,35,40,42,44], and one study was at high risk of bias for two domains with a risk of reporting bias for one domain [38]. High risk of performance bias was identified in one study [38], a moderate risk of performance bias was identified in twelve studies [30,32,33,34,35,36,39,40,42,43,44,46], and a low risk of performance bias was identified in three studies [29,31,41] .

### 2.9. Meniscal Repair

Two RCTs [47,48] and five non-randomized studies [49,50,51,52,53] evaluated the use of PRP in meniscus healing. In five studies, PRP was injected after arthroscopic meniscus repair [47,49,50,52,53], in one study patients underwent open meniscal repair with an adjunct of PRP [51], and in another the authors compared percutaneous meniscal trephination with or without PRP [6]. 

**Figure 9:**
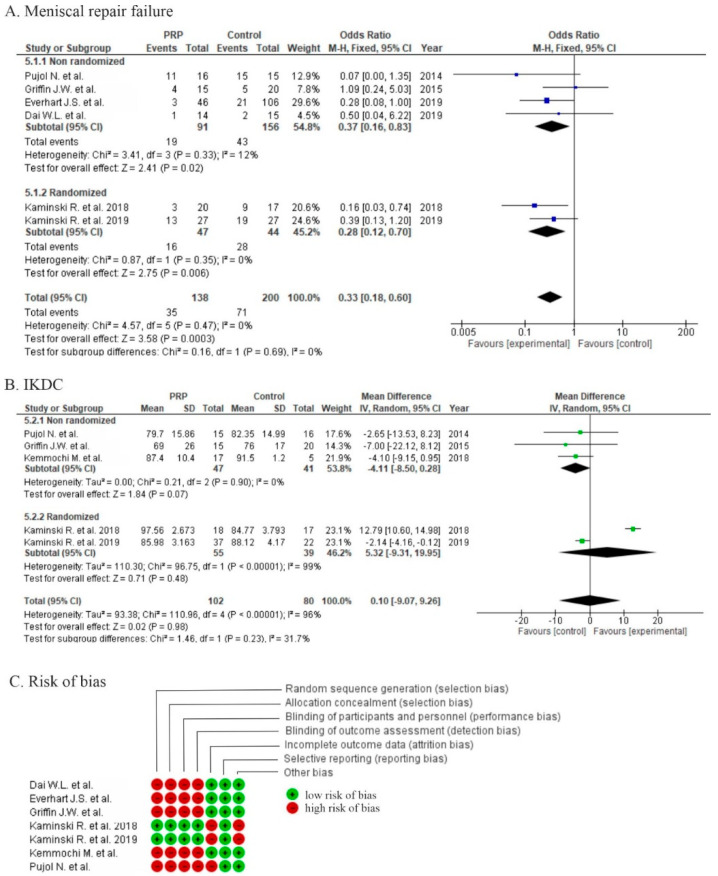
Forest plot for (**A**) meniscal repair failure and (**B**) IKDC (CI: confidence interval; IV: inverse variance; SD: standard deviation). (**C**) Risk of bias analysis.

Six studies reported failure rates of meniscus healing, two randomized studies using MRI and second-look arthroscopy showed significant differences in favor of PRP (*p* = 0.006) [47,48], and another four non-randomized studies also showed significant differences in favor of PRP (*p* = 0.02) [50,51,52,53]. In three studies the failure rate was defined by the need for revision surgery [50,52,53] and in the final study, MRI was used to assess meniscus healing [51]. One study did not provide any objective radiographic outcomes, only commenting “some” MRIs [49]. The pooled estimates for all six studies showed significant differences in favor of PRP (*p* = 0.0003), but due to the diversity of clinical trial types, synthesis provided only level of evidence III type data (retrospective cohort studies) with low heterogeneity (I^2^ 12%) (Figure 9A). Only one study reported outcomes after meniscus repair with concomitant ACLR, and the authors concluded that PRP healing effect depended upon the ACLR [52].

Five studies [47,48,49,50,51] reported functional results via IKDC scores; the pooled estimates for these studies showed non-significant differences in favor of the control (*p* = 0.98), although two randomized trials [5,6] showed non-significant differences in favor of PRP (*p* = 0.48) (Figure 9B).

Functional outcome was also recorded by the Lysholm score in three studies [49,50,53], by the KOOS score in three studies [47,48,51], and by the Tegner score in two studies [49,50]. 

Unfortunately, there is a large variety of clinical trial designs in this section, which may introduce a higher percentage of heterogeneity. Additionally, there could be an increase in heterogeneity via the study of Kaminski et al. [47] as the final assessment was made by two different methods. There is a strong need for more RCTs allowing for the performance of meta-analysis with low heterogeneity.

One study was at high risk of bias for five domains [51], four studies were at high risk of bias for four domains [49,50,52,53], and two studies were at high risk of bias for two domains [47,48]. High risk of performance bias was identified in five studies [49,50,51,52,53] and moderate risk of performance bias was identified in two studies [47,48] (Figure 9C).

### 2.10. Osteoarthritis

Thirty-eight studies, including 2962 patients, evaluated the use of PRP in osteoarthritis treatment. Thirty-three articles included patients with Kellgren–Laurence radiographic classifications system [54,55,56,57,58,59,60,61,62,63,64,65,66,67,68,69,70,71,72,73,74,75,76,77,78,79,80,81,82,83,84,85], five studies included patients with Ahlbäck radiographic classification system [86,87,88,89,90], and one study did not specify the osteoarthritis grade [91] (Table 8 and Table 9). Follow-up ranged from 6 months up to 2 years; thus, for such a large group as OA, heterogeneity will be too high due to our inability to compare outcomes at the same time point.

Twenty-eight studies compared PRP versus control groups [54,55,56,57,58,59,60,61,62,63,64,65,66,67,68,69,73,76,77,78,79,82,83,86,87,88,89,91], five studies compared PRP with the addition of another substance (MSC or HA) versus the control groups [74,79,81,91], six studies compared multiple injections of PRP [54,59,80,85,86,87], three studies compared PRGF-Endoret versus control groups [71,75,90], two studies compared autologous conditioned plasma (ACP) versus control groups [72,84], and one study compared intraosseous injection versus intra-articular injection versus the control group [67]. In twenty-three studies, HA [54,60,61,62,63,65,66,67,68,69,70,71,76,77,78,79,82,83,84,87,90,91] was used as a control, in ten studies placebo was used as a control (saline, no injection, physical therapy) [54,56,57,73,79,80,85,86,88,91], in four studies corticosteroids [58,59,74,77] were used as the control, and in two studies acetaminophen [55,64] was used as the control.

Thirty-three trials were included in the meta-analysis and another five were excluded due to being non-blinded [64,77,82,83,84].

**Figure 10:**
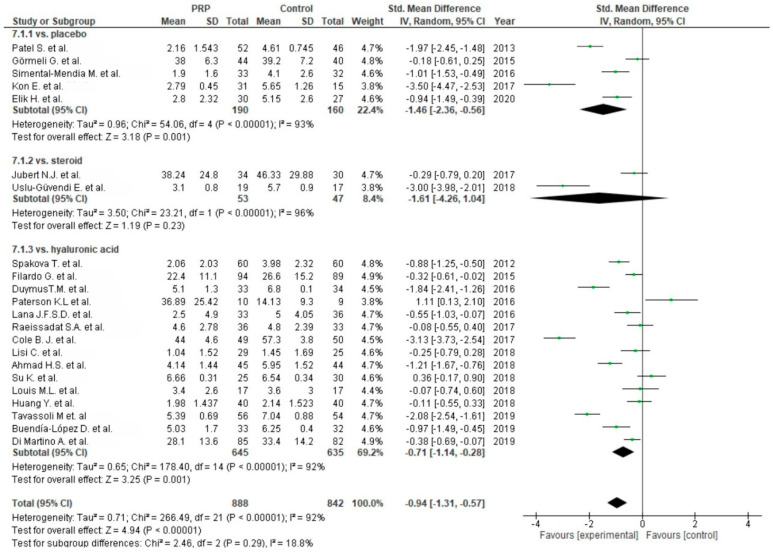
Forest plot for VAS comparing platelet-rich plasma (PRP) versus control (CI: confidence interval; IV: inverse variance; SD: standard deviation).

**Figure 11:**
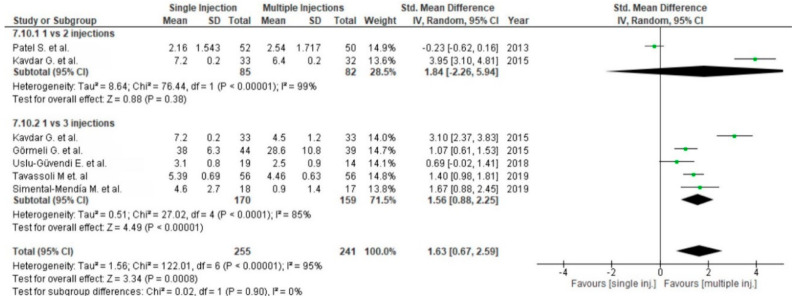
Forest plot for VAS comparing single versus multiple injections of PRP (CI: confidence interval; IV: inverse variance; SD: standard deviation).

Twenty-three studies reported pain via the VAS comparing PRP versus placebo [54,55,56,57,69,86], corticosteroids [58,59,77] or HA [54,60,61,62,63,65,66,67,68,69,70,71,76,77,78,79,87] (Figure 10). Placebo and HA subgroups showed significant differences in favor of PRP (*p* < 0.00001), despite the steroid subgroup showing non-significant differences in favor of PRP (*p* = 0.23). The pooled estimates for these studies also showed significant differences in favor of PRP (*p* < 0.00001). Six studies comparing single versus multiple (two or three times) injections of PRP assessed significant differences in favor of multiple injections (*p* = 0.0008) (Figure 11). However, only three injections of PRP showed significant differences compared to a single injection (*p* < 0.00001).

**Figure 12:**
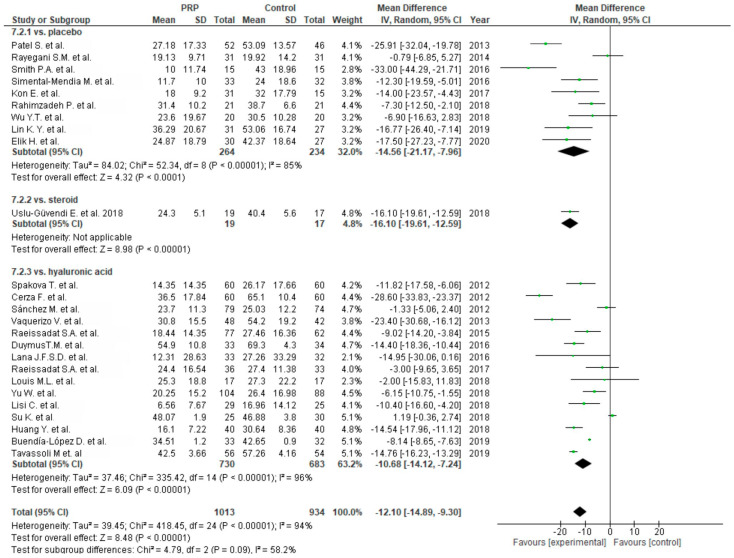
Forest plot for WOMAC scores (CI: confidence interval; IV: inverse variance; SD: standard deviation).

**Figure 13:**
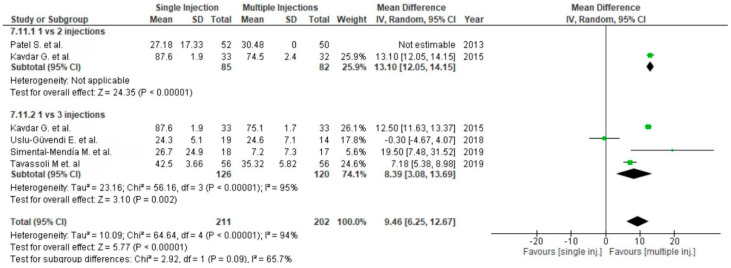
Forest plot for WOMAC scores comparing single PRP injection versus multiple PRP injections (CI: confidence interval; IV: inverse variance; SD: standard deviation).

Functional outcome was measured in twenty-eight studies via the WOMAC scale. One study was excluded from meta-analysis due to the reporting of only WOMAC pain scores [63]. Twenty-five studies compared PRP versus control groups: placebo [55,56,57,64,69,72,73,86,88,89,91], corticosteroids [59,77] or HA [62,63,65,67,68,69,71,75,77,79,82,83,84,87,89,90,91] (Figure 12). The pooled estimates for these studies showed significant differences in favor of PRP (*p* < 0.00001); furthermore each subgroup showed significant differences in favor of PRP (*p* < 0.00001). Functional outcomes were also analyzed in five studies [59,80,85,86,87] comparing single versus multiple injections and showed significant differences in favor of multiple injections (*p* < 0.00001), both in all studies and subgroups (Figure 13). 

**Figure 14:**
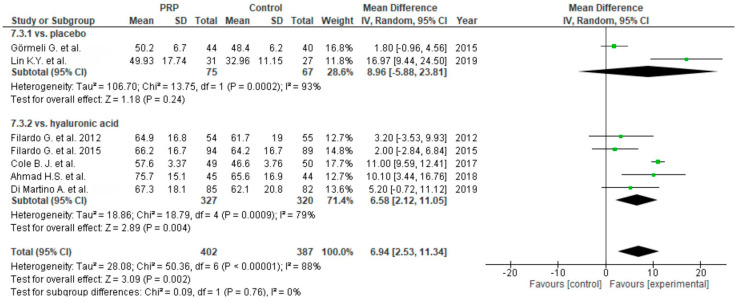
Forest plot for IKDC scores (CI: confidence interval; IV: inverse variance; SD: standard deviation).

Six studies evaluated functional outcomes in IKDC rating scores [54,60,63,66,70,78,89] and showed significant differences in favor of PRP (*p* = 0.002) (Figure 14). Five studies showed significant differences in favor of PRP compared to HA as a control group (*p* = 0.004) [60,63,66,70,78], and two studies showed non-significant differences in favor of PRP when compared with placebo (*p* = 0.24) [54,89]. 

**Figure 15:**
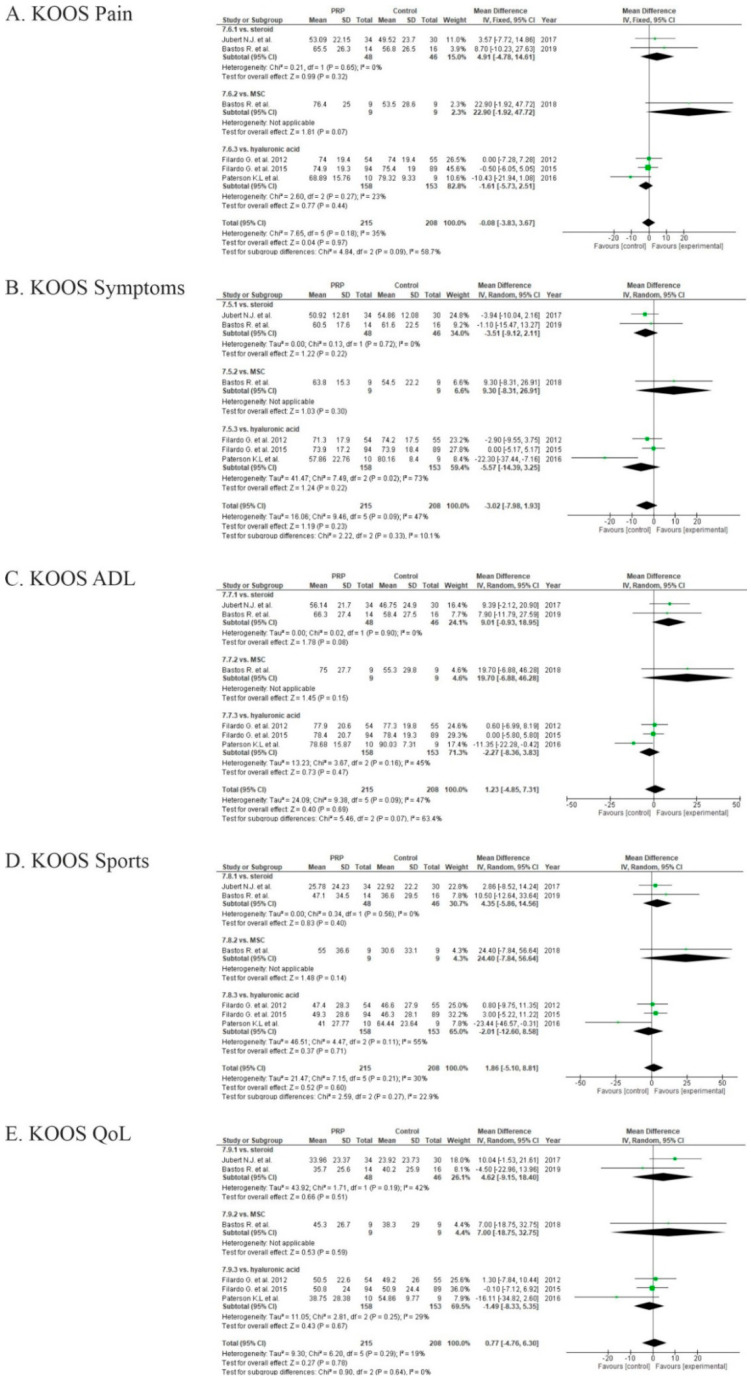
Forest plot for Knee injury and Osteoarthritis Outcome Score (KOOS) sub-scores: (**A**) pain; (**B**) symptoms; (**C**) activities of daily living (ADL); (**D**) sports; (**E**) quality of life (QoL). (CI: confidence interval; IV: inverse variance; SD: standard deviation).

Eight studies evaluated osteoarthritis outcomes via KOOS scores [56,58,60,61,74,76,78,81] (Figure 15). We excluded from the meta-analysis two of these studies, due to the lack of measurements in one [56] and division of the results according to the physician in another [76]. The pooled estimates for these studies showed non-significant differences in KOOS sport (*p* = 0.60), quality of life (*p* = 0.78), and ADL (*p* = 0.69) sub-scales in favor of PRP (*p* > 0.05), but in KOOS symptoms (*p* = 0.23) and pain (0.97) sub-scales were in favor of the control groups (*p* > 0.05).

Functional outcomes were also measured with the KSS score in one study [68], Lysholm score in two studies [63,68], Tegner score in four studies [60,68,70,79], Outcome Measures in Arthritis Clinical Trials–Osteoarthritis Research Society International (OMERACT–OARSI) pain measure in three studies [56,75,90], and Lequesne score in five studies [59,68,71,75,90]. Quality of life was measured with SF-36 scores in five studies [56,57,64,82], SF-12 in two studies [55,76], and European Quality of Life (EQoL) in two studies [61,76]. Significant improvement was shown in all KSS and Lysholm scores (*p* < 0.05), in 3/5 studies for the Lequesne score [59,68,75], and 1/3 study for OMERACT–OARSI scores [75]. No study detected significant difference in Tegner scores.

**Figure 16:**
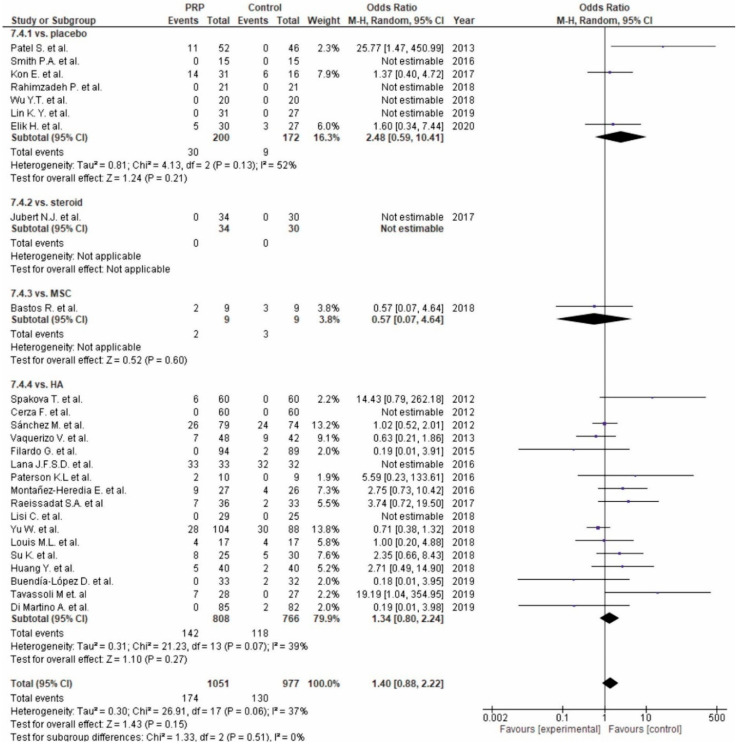
Forest plot for adverse events (CI: confidence interval).

Twenty-six studies reported adverse events (Figure 16). Seven studies [56,57,72,73,86,88,89] comparing PRP versus placebo reported non-significant differences in favor of the control groups (*p* = 0.21), fourteen studies [60,61,65,67,68,69,70,71,75,76,77,79,83,84,87,90,91] comparing PRP versus HA reported non-significant differences in favor of the control groups (*p* = 0.27), one study [58] comparing PRP versus steroids reported no adverse events [81], and one study comparing PRP versus MSC showed non-significant differences in favor of PRP (*p* = 0.60) [81]. The pooled estimates for these studies showed non-significant differences in favor of the control groups (*p* = 0.15).

**Figure 17:**
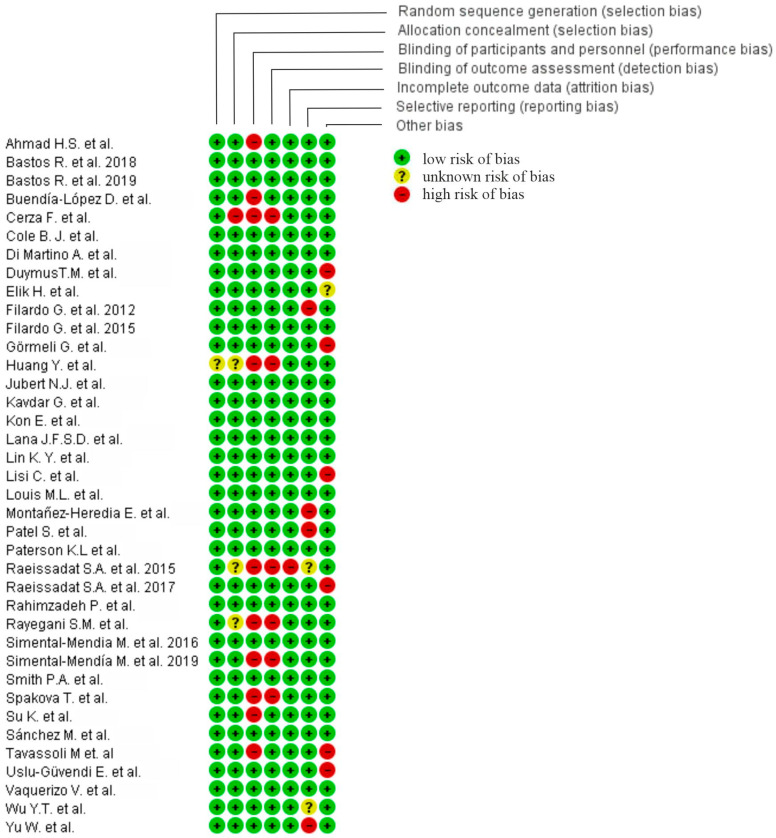
Risk of bias analysis for PRP application in osteoarthritis.

One study was at high risk of bias for three domains [84], two studies were at high risk of bias for two domains [83,85,87], and twelve studies were at high risk of bias for one domain [54,59,62,66,67,68,69,71,76,78,86,91]. One study was at high risk of performance bias for three domains with the risk of reporting bias for two domains [82], one study was at high risk of performance bias for two domains with risk of reporting bias for two domains [77], one study was at high risk of performance bias for two domains with risk of reporting bias for one domain [64], and two studies were at risk of reporting bias for one domain [57,88]. Low risk of performance bias was identified in seventeen studies, moderate risk of performance bias was identified in seventeen studies, and high risk of performance bias was identified in four studies (Figure 17).

## 3. Discussion

In recent years, blood derived products have been gaining more popularity in orthopedic treatment—especially platelet-rich plasma—due to their mechanism of action leading to stem cell proliferation, modulation of inflammatory processes, and angiogenesis [92]. It has resulted in an increased number of publications regarding the use of PRP in both conservative and intraoperative treatment, including systematic reviews and meta-analysis. However, this meta-analysis is the first concerning PRP applications in all knee diseases. 

The most important finding of our study was that PRP has some benefits in almost all analyzed subgroups. PRP improves outcomes in osteoarthritis applications, as well as in arthroscopic treatment of cartilage degeneration. PRP also has an influence on meniscus healing, faster return to sport after muscle injuries, and reduces blood loss after total knee replacement. 

Dupley et al. included two RCTs in their meta-analysis comparing PRP injection to ESWT or dry needling. They reported no significant differences in mean VISA-P scores at early follow-up (two or three months; difference in means, 11.9; standard error (SE), 7.4; 95% CI (–2.7, 26.4); *p* = 0.109). However, PRP was statistically better than the control at longer assessment periods (at six months or more than six months; difference in means, 12.7; SE, 4.4; 95% CI (4.1, 21.3); *p* = 0.004) [93]. Chen et al. included 11 studies in their meta-analysis, but only two RCTs [7,8], the same as Dupley et al. [93], both comparing the application of PRP to control in VISA-P and VAS. The mean difference in functional outcome was 13.22 (95% CI (2.37, 24.07)). In the pain scale, the mean difference comparing PRP with control groups was −1.87 (95% CI (−3.28, −0.46)) and showed that leukocyte-rich PRP (LR-PRP) has better functional improvement and pain reduction for patellar tendinitis compared with corticosteroids, treatment ultrasound, autologous blood injection (ABI) or topical glyceryl trinitrate (TGT) compared to control groups [94]. In our meta-analysis, four RCTs were included [6,7,8,9]. The results showed no significant differences in VAS (*p* = 0.78, 95% CI −0.17 (−1.38, 1.04)) and VISA-P scores (*p* = 0.97, 95% CI 0.52 (−11.50,12.54)). Two studies [8,9] provided better outcomes in both functional and pain scales. This may be the result of two injections of PRP over two weeks compared with a single injection in other studies [6,7]. Further research using a higher number of subjects and with lower biases is needed to state unequivocally that the number of injections positively influences the effect of PRP on PT treatment.

Grassi et al. performed a meta-analysis evaluating outcomes after PRP application in acute muscle injuries [95]. Six RCTs showed significantly shorter time for return to sport in the PRP group (*p* = 0.006, 95% CI −7.17 (−12.26, −2.08)); however, in three studies with hamstring injuries the difference was not significant (*p* = 0.07, 95% CI −5.95 (−12.48, 0.57)). No other significant differences for fixed-effect meta-analysis among the group were found including re-injury rate, complications, pain, muscle strength, function, ROM, and imaging. Three studies reported better pain outcomes in the PRP group (*p* < 0.05). Bubnov et al. reported a greater ROM and higher strength in the PRP group [96]. In our review we analyzed four RCTs which included hamstring injuries. Time for return to sport was significantly shorter in PRP versus control groups (*p* < 0.00001, 95% CI −4.16 (−5.44, −2.88)). The differences in hamstring meta-analyses may be the result of a narrow range of 95% CI in one additional study (−3.90 (−5.27, −2.53)) or it could be the result of the evaluation of other muscle injures except the hamstring (quadriceps, gastrocnemius) [10]. We also failed to find any significant differences in re-injury rate (*p* = 0.50). In two studies we also reported lower pain severity. However, more prospective studies for PRP application after muscle injuries are needed as current research shows promising results for a faster return to sport, which can be a major advantage especially for athletes. 

In our synthesis we included only two RCTs with PRP application in high tibial osteotomy; this was due to a lack of similar studies. Both RCTs evaluated different outcomes, with significant differences in functional and pain scales, as well as in radiological bone healing and second-look arthroscopy cartilage healing. Roffi et al. performed a systematic review on the application of PRP in bone healing, identifying forty-five pre-clinical in-vivo studies and nineteen clinical studies. Nine clinical studies addressed the role of PRP in the treatment of fractures. Six of them showed improved results in PRP groups regarding radiological parameters. Only five trials reported functional outcomes, with two studies providing improved outcomes. Another ten studies addressed the treatment of delayed or non-unions. Eight of them suggested a positive role for PRP in stimulating bone healing [97]. The results are promising; however, further research is necessary to confirm the effectiveness of PRP in accelerating bone healing and to exclude bias. Good outcomes in these two studies could be the result of an addition of other myeloid stromal cells. 

Muchedzi et al. included seventeen RCTs for evaluation of PRP in both osteoarthritis and following total knee arthroplasty. Primary outcomes after TKA were presented in five studies and included less pain in short-term follow-up in the PRP group (*p* = 0.05, heterogeneity 91%), but no improvements in functional outcome in WOMAC scores. Secondary outcomes were evaluated in ten studies with no significant differences in blood loss (*p* = 0.07). Three studies provided no benefits in length of hospital stay (*p* = 0.31) [98]. We included six RCTs and analyzed blood loss after TKA to show significant reduction in blood loss in the PRP group (*p* = 0.001, 95% CI −0.29 (−0.46, −0.11)). Four studies reported better outcomes in VAS in the PRP group in short-term follow up. None of the studies showed differences in functional outcomes or range of motion. The differences in outcomes may be a result of study choice as we analyzed only RCTs. Promising results in decreasing pain and blood loss after TKA should encourage further well-planned RCTs with a higher number of patients. 

There exists no previous meta-analysis evaluating the use of PRP in addition to arthroscopic surgery. Good outcomes in cartilage healing after PRP injection to knee joint is known [99]. This suggests that the addition of PRP to surgical treatment might also have a satisfactory effect. Comparing microfractures to PRP injection showed significantly better outcomes in IKDC and Lysholm scores (*p* < 0.00001; *p* = 0.03) for the PRP group, although the results are only level of evidence III. The positive effects of microfractures in arthroscopic surgery are known, so further extensive research for arthroscopy with concomitant PRP treatment should be encouraged.

Davey et al. analyzed anterior cruciate ligament reconstruction with augmentation of PRP. Thirteen RCTs showed neither significant improvement in any of the clinical outcomes (Tegner, Lysholm, KOOS, IKDC) nor in pain reduction (*p* = 0.18). PRP also does not support graft healing or donor-site morbidity [100]. In our study including 16 RCTS, we also did not find significant improvements in functional outcomes (IKDC, Lysholm, Tegner), pain reduction (VAS), stability assessment (KT-1000) or tunnel widening. Every outcome crosses the zero line in the forest plot. Currently there is no evidence for supporting ACLR by PRP injection, despite the numerous positive effects of PRP in other diseases. 

Haunschild et al. performed a systematic review, including five studies (two prospective and three retrospective) comparing PRP augmentation of meniscus repair to meniscus repair alone. Three studies showed no significant differences in outcome or failure, another two had improvements at the final follow up (KOOS, IKDC, WOMAC, failure). Three studies assessed radiographic findings using MRI: Pujol et al. [51] showed a significantly improved healing rate (*p* < 0.01); Kaminski et al. [47] showed insignificant findings on MRI, although significant improvement was reported in second-look arthroscopy; and Kemmochi et al. [49] failed to show clearly any improvement only revealing a tendency toward healing. We included in the meta-analysis two additional studies (two RCTs and five non-randomized). Six studies of meniscal repair failure reported significant differences in favor of the PRP group (*p* = 0.003, 95% CI 0.33 (0.18; 0.60)), one study [49] was excluded because of unclear criteria of improvements in a follow-up MRI and lack of exact outcomes. When comparing with previous analysis where improvements in failure rate were not clearly demonstrated, it is probable that we added two more studies with high weight in our meta-analysis (29.6%, 24.6%). Six studies reported functional outcomes, but only three of them reported significant improvements in some of the scores (KOOS, IKDC, WOMAC). There is insufficient evidence for the addition of PRP to meniscus repair treatment, however, we conclude there are some promising results which should encourage more randomized clinical trials in the near future.

Zhang et al. performed a systematic review of thirteen studies (ten RCTs, three prospective) comparing PRP application in osteoarthritis versus hyaluronic acid. Pain outcomes estimated by VAS did not reveal significant differences and WOMAC pain was significantly decreased after 6 and 12 months of follow-up (*p* < 0.01; mean difference (MD = −15.25; 95% CI: −22.17 to −8.32). In addition, WOMAC physical function showed significant differences in favor of the PRP group (*p* < 0.01; MD 11.17; 95% CI (–16.37, –5.98). Functional outcomes in IKDC scale was significant at 6 months of follow-up (*p* < 0.01), however differences were not significant among the groups after 12 months (*p* = 0.13) [101]. Similarly, Vilchez-Cavazos et al. evaluated the treatment of knee osteoarthritis comparing a single PRP injection versus multiple PRP injections. Five RCTs measured pain and functional outcomes showing insignificant differences in favor of multiple injections in pain scores (*p* = 0.19; 95% CI 0.65 (−0.31; 1.60)) but significantly better results on joint function in the WOMAC score when comparing multiple injections versus single injection (*p* < 0.0001; 95% CI 2.24 (1.12, 3.36)) [102]. Our analysis of PRP injection in the treatment of an osteoarthritic knee included thirty-eight studies. Hyaluronic acid, corticosteroids, saline, no injection, and acetaminophen were evaluated as control groups. The most significant conclusion was that multiple injections were significantly more effective than a single injection with respect to pain (VAS, *p* = 0.0008, 95% CI 1.63 (0.67; 2.59)) and functional outcomes (WOMAC, *p* < 0.00001, 95% CI 9.46 (6.25; 12.67)). However, we did not find any correlation between injection intervals and clinical outcomes. PRP application was repeated after 1, 2, 3 or 4 weeks. Only one study assessed the effects of a different number of PRP injections. Kavadar et al. compared one, two, and three injections of PRP in grade 3 OA. Mean differences in VAS and TUG (Timed Up and Go Test) significantly favored multiple injections (1 inj. vs. 2 inj., 1 inj. vs. 3 inj., and 2 inj. vs. 3 inj.), but WOMAC mean differences were significant only in comparison of single versus multiple injections [80]. There is a strong need for more RCTs evaluating the effectiveness of multiple injections of PRP and answering the question: Is twice the applications of PRP injection satisfactory for an optimal clinical effect or does effectiveness improve with the number of injections? Our significant results in pain outcomes derive from the accurate VAS measurements; Vilchez-Cavazos used change in pain data for comparison, despite the fact that even a single injection of PRP significantly improved pain (*p* < 0.00001) and functional outcomes in WOMAC and IKDC (*p* < 0.00001; *p* = 0.002) versus control groups. Only in KOOS scores were differences not significant for pooled estimates studies. This might be a result of the small group of included studies. Furthermore, 50% of these studies (3/6) included hyaluronic acid as a control group which was the only one showing insignificant differences in favor of control groups. Some authors concluded that this could be the reason for using LR-PRP or an older population of patients [60]. The analysis of adverse events also showed the advantage of PRP over other treatments. Differences were not significant but still PRP seems to be the safer option for patients (*p* = 0.15; 95% CI 1.40 (0.88, 2.22)). 

There is no single method of PRP preparation and there are many devices and protocols being used. In our synthesis, the most frequently applied centrifuge was the GPS System III (Zimmer Biomet). In addition, there is no evidence for improved outcomes after leukocyte addition to PRP. Most of the studies used leukocyte-rich platelet-rich plasma. There were only two studies in our metanalysis comparing Leukocyte-poor and leukocyte-rich PRP. The first, in the patellar tendinopathy section, showed a non-significant difference in VISA and VAS scores in favor of leucocyte-rich PRP [6]. A second study which compared PRGF (leukocyte poor) with leukocyte-rich PRP, showed only a significant difference in swelling scores on the first day and CRP ten days after surgery in favor of PRGF. There is a lack of studies comparing leukocyte concentration in PRP to clinical outcomes. Hanish et al. did not find any significant differences between leukocyte-poor (LP)-PRP and LR-PRP in treatment of Achilles tendinopathy in VISA-A and VAS [103]. Further, Yerlikali et al. showed no significant differences in pain, functional parameters, and inflammatory reaction between LR-PRP and LP-PRP in patients with lateral epicondylitis [104]. Riboh et al. performed a meta-analysis including six RCTs and three prospective comparative studies comparing efficacy of leukocyte concentration in OA treatment. The final analysis included WOMAC score, IKDC score, and adverse events. They showed a slight advantage in functional outcomes favoring LP-PRP, but leukocyte concentration did not influence upon adverse reactions [105]. Despite the small amount of studies, leukocytes may be important factors in supporting the action of PRP. They play a role in regeneration through stimulation of immune processes. Lana et al. suggested leukocyte-rich PRP may have some benefits over leukocyte-poor PRP, due to macrophage inclusion, which are “like instructors of the healing orchestra”, because of their role in remodeling and repair phases [106]. On the other hand, Braun et al. concluded that leukocytes provide acute inflammatory response and LP-PRP leads to better outcomes of synovial cell treatment than LR-PRP [107]. There is a strong need for further research on the effectiveness of LP and LR-PRP. 

Our metanalysis showed and summarized many positive effects of PRP. However, there are still many unsolved questions and issues requiring specific studies that should be performed according to the “DOSES” cell-therapy communication tool [108]. This standardized system for describing cell therapies allows the systematic performance of RCTs and full clinical outcome assessment of PRP in knee disorders.

### 3.1. Strengths

Our major strength is that this synthesis includes comprehensive analysis of PRP application in the treatment of major knee lesions. We performed level of evidence I analysis in all types of lesions but two, which included level of evidence III studies. Our promising results could be a result of a large amount of included RCTs and wide array of control groups. This reduces the risk of bias and provides a more complete and reliable analysis.

### 3.2. Limitations

English language trials were included, but non-English language studies were excluded; they also may contain relevant research. The PRP preparation kits were heterogeneous and not always clearly defined, furthermore platelet count and leukocyte content differed. Level of evidence of included studies varied (I–II). Five of seven included studies in one subgroup were non-randomized (level of evidence III). Some studies with a high risk of bias could have influenced the final results of this synthesis. The diversity of scales used did not allow us to perform meta-analysis of every outcome. Additionally, some of the syntheses consisted of high heterogeneity studies (above 40%).

## 4. Materials and Methods

### 4.1. Search Strategy

In this review we concentrated on PRP application in knee lesions compared with placebo- or other treatment control groups. This study was completed in compatibility with the 2009 Preferred Reporting Items for Systematic Reviews and Meta-Analyses (PRISMA) statement. A systematic review of the use of platelet-rich plasma in knee lesions was completed with a comprehensive published literature search through PubMed, Embase, Cochrane Database of Systematic Reviews, and Clinicaltrials.gov. The references of the investigations found in this search were cross-referenced to identify additional pertinent studies not identified in the original searches. All searches were performed in February 2020. The searches were performed combining the following keywords: (1) “PRP” or “platelet-rich plasma” or “plasma rich in growth factors” or “platelet derived growth factor” or “platelet derived” or “platelet gel” or “platelet concentrate” or “PRF” or “platelet rich fibrin” or “ACP” or ”autologous conditioned plasma” or ”PRGF” or “platelet lysate”, and (2) “knee” or “knee osteoarthritis” or “meniscus” or “menisci” or “chondral” or “cartilage” or “ligament” or “patella” or “patellar” or “PCL” or “MCL” or “iliotibial” or “osteochondritis” or “hamstring” or “quadriceps” or “epicondyle” or “osteonecrosis” or “arthroscopy” or “tibia” or “tibial” or “femur” or “femoral” or “trochlea” or “posterolateral” or “posteromedial” or “chondrocyte” or “articular” or “arthroplasty” or “osteotomy” or “red zone” or “white zone” or “extrusion” or “red-white” or “intra-meniscal”. Systematic review registration was performed on 10.02.2020 using PROSPERO (International Prospective Register of Systematic Reviews, ID 167715).

### 4.2. Inclusion and Exclusion Criteria

This review included all clinical studies meeting the following inclusion criteria: PRP utilization as conservative treatment in knee lesions or as support in knee surgery, English language, human subjects, paper published in a peer-reviewed journal, and full text available. Only randomized controlled trials were included, in addition to the meniscus and microfractures section where only a small number of RCTs was identified. Exclusion criteria included all animal studies, basic scientific investigations, case reports, review articles, expert opinions, letters to editor, studies without control groups, studies not using PRP, papers not peer reviewed, papers not in English, trials evaluating platelet-poor plasma, and investigations on other diseases unrelated to the knee joint. The investigations included in this study were independently reviewed by two orthopedic surgeons/authors for inclusion and exclusion criteria. 

### 4.3. Types of Interventions

We compared intralesional, injected PRP preparation with:

- placebo injection (low volume saline injection, matching the prp volume);
- high volume saline image guided injection;
- local steroids injection;
- hyaluronic acid injection;
- exercise and other physical therapies (e.g., low-dose radiation therapy, eccentric loading program, dry needling);
- any other medications given locally or systemically aimed at treating pain; and
- combinations of the active interventions listed above.

### 4.4. Outcomes

Primary outcomes included:

- pain as measured by standard validated pain scale, such as visual analogue score (VAS), EQ-VAS or numerical rating scale (NRS);
- functional measurement by any standard validated scale, such as the International Knee Documentation Committee (IKDC), Western Ontario and McMaster Universities Osteoarthritis Index (WOMAC), Knee Society Score (KSS), Victorian Institute of Sport Assessment (VISA), 36-Item Short Form Survey (SF-36), Knee injury and Osteoarthritis Outcome Score (KOOS), Lysholm Knee Scoring Scale, Teger Activity Score, and Ikeuchi grade knee rating scale;
- meniscal repair failure;
- time for return to sport;
- re-injury;
- knee stability, measured as tibial translation;
- graft integration; and
- tunnel widening.

Adverse events were also evaluated and analyzed. If multiple time points were reported within our time frames, we extracted the last time point (e.g., if data were reported at six weeks, three months, six months, and one year, we extracted outcomes at one year).

### 4.5. Data Collection and Analysis 

For each study included in the analysis, the following data were extracted by two independent reviewers: authors, year of publication, type of knee lesions, details of interventions in the study, sample size (randomized and analyzed), outcome measurements, follow-up period, main results, and percentage and type of adverse events included in the publication. Each study’s level of evidence was examined and evaluated based on criteria established by Oxford Centre for Evidence-Based Medicine Levels of Evidence Working Group [109]. Measures of treatment effect at a final point were the mean and standard deviation for continuous outcome measures. When studies reported other measures (e.g., median) and other dispersion measures such as standard error (SE) of the mean or 95% CI of the mean, range or interquartile range (IQR) we calculated the SD in order to perform the relevant meta-analytical pooling according to previous studies (see [110,111]). 

The study weight was calculated using the Mantel–Haenszel method. We assessed statistical heterogeneity using Tau^2^ or Chi^2^, df and I^2^ statistics. The I^2^ statistic describes the percentage of total variation across trials that is due to heterogeneity. In the case of low heterogeneity (I^2^ < 40%), studies were pooled using a fixed-effects model, otherwise a random-effects analysis was made.

Subgroup analysis was undertaken for the type of clinical trial and for the type of control intervention.

### 4.6. Assessment of Risk of Bias

Revised Cochrane risk-of-bias tool was used to evaluate risk. Disagreement in the risk of bias assessment was resolved by consensus and, if necessary, by the opinion of a third reviewer. A study was deemed to be:

- “low risk” if all items were scored as “low risk”
- “moderate risk” if up to two items were classified as “high risk” or “unclear risk”
- “high risk” if more than two items were scored as “high risk”

We presented our assessment of risk of bias using two “Risk of Bias” summary figures for every sub-section of the manuscript. 

### 4.7. Statistical Analysis 

Qualitative statistical analysis and meta-analysis were performed using R software and REVMAN 5.3 [112,113] with *p*-values of < 0.05. 

## 5. Conclusions

Our systematic review and meta-analysis of the clinical use of platelet-rich plasma in knee lesions show some promising results. First of all, our study confirms significant benefits in the use of PRP in osteoarthritis compared with various control groups. PRP is also safe for patients when compared to control groups; there is an insignificant difference in adverse events in favor of control groups. In other subgroups, differences in functional or pain scales or other measured parameters were significant in favor of the PRP group (blood loss in TKA, time to return to sport in hamstring injuries, microfractures augmentation). Some analyzed results showed an advantage of PRP compared to control groups and should lead to further research with a higher number of subjects and with lower biases to state unequivocally that PRP is a necessary component of the treatment (meniscal repair failure). Only one area clearly showed no differences in ACL reconstruction with or without PRP, thus we conclude so far that PRP has not been proved beneficial in ACLR. However, we do recommend PRP application in knee osteoarthritis and suggest performing more clinical trials concerning PRP application in other knee lesions. The optimal protocol (e.g., number of injections, timeframe) for the most effective treatment should be determined. Methods of preparation of platelet-rich plasma need further standardization. Studies should be performed to establish adequate cost–benefit of PRP compared with other standard, less expensive, treatments. 

## Figures and Tables

**Table 1:** Platelet-rich plasma (PRP) compared with control intervention for patellar tendinopathy.

	LOE	Type of Study	Exp	Cont	Follow-up	Control	Preparation Kit	LR/LP	Platelet Conc.	Number of Inj.	PROM	Ref.
Abate et al.	III	3 arms	PRP 18(18) HVIGI + PRP 18(18)	18(18)	6 months	HVIGI saline	Regen Lab A-PRP Kit (Regenlab)	LP	1.6× NPC (Native Platelet Concentration)	2	VISA VAS	[9]
Dragoo et al.	I	2 arms	10(8)	12(9)	6 months	Dry needling	GPS III (Biomet)	LR	N/R	1	VISA Tegner Lysholm VAS SF-12	[7]
Scott et al.	I	3 arms	LR 19(19) LP 19(19)	19(19)	12 months	Saline	ACs (Arthrex)	LR/LP	LR 3.8 × 230,000 (51,000)/µL LP 3.0 × 227,000 (43,000)/µL	1	VISA NPRS GROC	[6]
Vetrano et al.	I	2 arms	23(23)	23(23)	12 months	ESWT	Recover ps kit (Kaylight)	N/R	0.89–1.1 × 10^9^ µL	2	VISA VAS Blazina	[8]

LOE—level of evidence; exp.—no. of patients receiving treatment in experimental group (no. of patients analyzed at final follow-up); cont.—no. of patients receiving treatment in control group (no. of patients analyzed at final follow-up); ESWT—extracorporeal shock wave therapy; HVIGI—high volume image guided injection; LR—leukocyte rich; LP—leukocyte poor; PROM—patient related outcome measures; VAS—visual analog scale; NPRS—Numeric Pain Rating Scale; VISA—Victorian Institute of Sport Assessment; GROC—Global Rating of Change Scales; SF-12—Short Form Survey.

**Table 2:** Platelet-rich plasma (PRP) compared with control intervention for the knee adjacent muscle injuries.

	LOE	Type of Study	Exp.	Cont.	Follow-up	Control	Preparation Kit	LR/LP	Platelet Concentration	Number of Injections	PROM	Ref.
Hamid et al.	II	2 arms	14(12)	14(12)	39 weeks	No injection	GPS III (Biomet)	38.3 × 10^3^/µL	1297 × 10^3^µL	1	RTS BPI-SF	[11]
Hamilton et al.	I	3 arms	PRP 30(26) PPP 30(28)	30(29)	6 months	No injection	GPS III (Biomet)	26.1(13.7) × 10^3^/µL	765.8(23.6) × 10^9^/L	1	RTS Re-injury	[12]
Reurink et al.	I	2 arms	41(37)	39(36)	1 year	Saline	ACP (Athrex)	1.9(2.1) × 10^3/^µL	433(128) × 10^3^/µL	2	RTS Re-injury	[13]
Rossi et al.	I	2 arms	35(34)	40(38)	2 years	No injection	N/R	N/R	N/R	1	RTS VAS Re-injury	[10]

LOE—level of evidence; exp.—no. of patients receiving treatment in experimental group (no. of patients analyzed at final follow-up); cont.—no. of patients receiving treatment in control group (no. of patients analyzed at final follow-up); LR—leukocyte rich; LP—leukocyte poor; PROM—patient related outcome measures; VAS—visual analog scale; RTS—time for return to sports; BPI-SF—Brief Pain Inventory-Short Form.

**Table 3:** Platelet-rich plasma (PRP) compared with control intervention for high tibial osteotomy.

	LOE	Type of Study	Exp.	Cont.	Follow-up	Control	Preparation Kit	LR/LP	Platelet Concentration	Number of Injections	PROM	Ref.
Dallari et al.	I	3 arms	PG 1(9) PG + BM 12(10)	10(9)	1 year	Lyophilized bone chips	N/R	N/R	1 × 10^6^/µL	1	KSS ROM Osteointegration histomorphometric	[15]
Koh et al.	II	2 arms	26(23)	PRP + MSC 26(21)	2 years	PRP + MSC	N/R	N/R	1303.27 (375.2) × 10^3^/µL	1	Lysholm VAS KOOS	[14]

LOE—level of evidence; exp.—no. of patients receiving treatment in experimental group (no. of patients analyzed at final follow-up); cont.—no. of patients receiving treatment in control group (no. of patients analyzed at final follow-up); LR—leukocyte rich; LP—leukocyte poor; ROM—range of movement; PROM—patient related outcome measures; VAS—visual analog scale; KSS—Knee Society Score; Lysholm—Lysholm Knee Scoring Scale.

**Table 4:** Platelet-rich plasma (PRP) compared with control intervention for TKA.

	LOE	Type of Study	Exp.	Cont.	Follow-up	Control	Preparation Kit	LR/LP	Platelet Concentration	Number of Injections	PROMs	Ref.
Guerreiro et al. (2015)	I	2 arms	20(20)	20(20)	2 months	no injection	Fanem	LP	988,250	1	VAS WOMAC HgB drop ROM Ht Wound	[19]
Guerreiro et al. (2019)	I	4 arms	PRP 20(16) PRP + TXA 20(18)TXA 23(13)	21(21)	2 years	saline	Fanem	LP	618,500	1	VAS WOMAC HgB drop ROM Wound	[20]
Horstman et al.	I	2 arms	20(20)	20(20)	10 days	no injection	GPS (Biomet)	LR	N/R	1	VAS HgB drop ROM wound	[16]
Mochizuk et al.	I	2 arms	109	206	14 days	no injection	N/R	N/R	N/R	1	HgB drop ROM BL	[21]
Morishita et al.	I	2 arms	20(20)	20(20)	28 days	no injection	ACS (Exactech)	LR	23.4 × 104/µL	1	KOOS KSS HgB drop ROM BL CRP	[17]
Peerboom et al.	II	2 arms	50(32)	52(41)	3 months	no injection	GPS (Biomet)	N/R	N/R	1	VAS WOMAC HgB drop ROM wound	[18]

LOE—level of evidence; exp.—no. of patients receiving treatment in experimental group (no. of patients analyzed at final follow-up); cont.—no. of patients receiving treatment in control group (no. of patients analyzed at final follow-up); LR—leukocyte rich; LP—leukocyte poor; ROM—range of movement; BL—blood loss; wound—wound healing; CRP—C reactive protein; HgB—hemoglobin; Ht—hematocrit; PROM—patient related outcome measures; VAS—visual analog scale; WOMAC—Western Ontario and McMaster Universities Osteoarthritis Index; KSS—Knee Society Score; KOOS—Knee injury and Osteoarthritis Outcome Score.

**Table 5:** Platelet-rich plasma (PRP) compared with control intervention as adjunct treatment for arthroscopy.

	LOE	Type of Study	Exp	Cont	Follow-up	Control	Preparation Kit	LR/LP	Platelet Concentration	Number of Inj.	PROM	Ref.
Duif et al.	II	2 arms	24(21)	34(32)	12 months	no injection	ACP (Arthrex)	LP	N/R	1	VAS IKDC Lysholm SF-36	[23]
Kim et al. (2015)	III	2 arms	MCS + PRP 71(20)	94(20)	24 months	MSC + fibrin glue	Process Protocol	N/R	1.28 × 10^6^/µL	1	IKDC Tegner ICRS	[28]
Lee et al.	I	2 arms	24(24)	25(25)	24 months	microfracture	Magellan APS (MBTD)	N/R	N/R	1	VAS IKDC Lysholm	[24]
Manunta et al.	II	2 arms	10	10	12 months	microfracture	GPS II (Biomet)	N/R	N/R	3	VAS IKDC	[25]
Manco et al.	III	2 arms	14	13	24 months	microfracture	Manual	N/R	0.3–1.5 × 10^6^	1	VAS IKDC SF-36	[27]
Nguyen et al.	III	2 arms	15(15)	15(15)	18 months	microfracture	New-PRP Pro Kit (GeneWorld)	N/R	N/R	1	WOMAC VAS Lysholm Outerbridge	[26]
Vasavilbaso et al.	I	5 arms	10(10)	control 10(10) HA 3 10(10) HA 4 10(10) HA 5 10(10)	18 months	no injection HA	GPS II (Biomet)	N/R	N/R	1	WOMAC	[22]

LOE—level of evidence; exp.—no. of patients receiving treatment in experimental group (no. of patients analyzed at final follow-up); cont.—no. of patients receiving treatment in control group (no. of patients analyzed at final follow-up); LR—leukocyte rich; LP—leukocyte poor; ROM—range of movement; BL—blood loss; wound—wound healing; CRP—C reactive protein; HgB—hemoglobin; Ht—hematocrit; PROM—patient related outcome measures; VAS—visual analog scale; WOMAC—Western Ontario and McMaster Universities Osteoarthritis Index; SF-36—36-Item Short Form Survey; Outerbridge—Outerbrige cartilage injury scale; Lysholm—Lysholm Knee Scoring Scale; IKDC—International Knee Documentation Committee.

**Table 6:** Platelet-rich plasma (PRP) compared with control intervention as adjunct treatment for ACL reconstruction.

	LOE	Type of Study	Exp	Cont	Follow-up	Control	Preparation Kit	LR/LP	Platelet Concentration	Number of Injections	PROM	Ref.
Almeida et al.	I	2 arms	12(10)	15(12)	6 months	no injection	995-E (Haemonetics Corp, Braintree)	0.91/mm^3^± 0.81/mm^3^	1,185,166/mm^3^± 404,472/mm^3^	1	Kujala VAS IKDC Lysholm Tegner	[32]
Azcarate et al.	II	3 arms	50(50) PG 50(50) Endoret	50(50)	12 months	no injection	Beckman J-6B BTI System II	LP/LR	837 × 10^6^/mL 504 × 10^6^/mL	1	CRP VAS KT-1000 IKDC MRI	[34]
Cervellin et al.	I	2 arms	20(20)	20(20)	12 months	small blood sample	GPS II (Biomet)	LR	N/R	1	VAS VISA	[29]
Mirzatolooei et al.	I	2 arms	25(23)	25(23)	3 months	no injection	ACP (Arthrex)	LP	N/R	1	CT tunnel widening VAS ROM KT-1000	[41]
Orrego et al.	II	4 arms	PC 29(26) BP 29(28) PC + BP 29(27)	29 (27)	6 months	no injection	GPS II (Biomet)	LR	N/R	1	MRI graft maturation IKDC Lysholm	[39]
Radice et al.	III	2 arms	25	25	1 year	no injection	GPS (Biomet)	N/R	N/R	1	graft integration MR	[33]
Rupreht et al.	II	2 arms	25(21)	25(20)	6 months	no injection	N/R	N/R	978 × 10^3^/ mm^3^	1	tunnel healing MRI	[37]
Sanchez et al.	III	2 arms	22(21)	15 (15)	24 months	no injection	BTI System II	LP	2–3 × NPC	1	Histology—remodeling graft 2nd arthroscopy	[40]
Seijas et al. (2013)	I	2 arms	49(48)	49(48)	12 months	no injection	BTI System	N/R	N/R	1	MRI graft remodeling	[35]
Seijas et al. (2016)	I	2 arms	23	20	2 years	no injection	N/R	N/R	N/R	1	VAS	[31]
Silva et al.	I	4 arms	10 1xprp 10 3xprp 10 Clotalys	10	3 months	no injection	GPS III (Biomet)	LR	N/R	3	graft integration MR	[36]
Starantzis et al.	II	2 arms	30(25)	30(26)	1 year	placebo sample	GPS III (Biomet)	LR	N/R	1	MRI CT tunnel diameter Lysholm KT-1000	[42]
Sözkesen et al.	III	2 arms	18	26	12 months	no injection	Prosys PRS bio kit (Prodizen)	N/R	N/R	1	IKDC Lysholm Tegner KT-1000 CT tunnel healing	[43]
Vadala et al.	II	2 arms	20	20	10 months	no injection	PRP Fast Biotech kit (MyCells)	N/R	N/R	1	Tegner Lysholm IKDC KT-1000 CT tunnel enlargement	[44]
Ventura et al.	I	2 arms	10(10)	10(10)	6 months	No injection	GPS (Biomet)	N/R	N/R	1	KOOS IKDC KT-1000 Tegner	[38]
Walters et al.	II	2 arms	27(17)	23(12)	24 months	bone chips with no injection	ACP (Arthrex)	LP	2–3 × NPC (<750,000 platelets/µL)	1	VAS VAS ADL IKDC	[30]

LOE—level of evidence; exp.—no. of patients receiving treatment in experimental group (no. of patients analyzed at final follow-up); cont.—no. of patients receiving treatment in control group (no. of patients analyzed at final follow-up); LR—leukocyte rich; LP—leukocyte poor; ROM—range of movement; CRP—C reactive protein; MRI—magnetic resonance imaging; CT—computer tomography; PROM—patient related outcome measures; VAS—visual analog scale; WOMAC—Western Ontario and McMaster Universities Osteoarthritis Index; Tegner—Tegner Activity Score; Lysholm—Lysholm Knee Scoring Scale; IKDC—International Knee Documentation Committee.

**Table 7:** Platelet-rich plasma (PRP) compared with control intervention as adjunct treatment for meniscus repair.

	LOE	Type of Study	Exp	Cont	Follow-up	Control	Preparation Kit	LR/LP	Platelet Concentration	Number of Inj.	PROM	Ref.
Dai et al.	III	2 arms	14(13)	15(13)	1 year	no injection	N/R	LR	6.4 ± 1.6 ×NPC	1	Lysholm Ikeushi VAS Failure	[53]
Everhart et al.	III	3 arms	203(164) 148 55	347(294)	3 years	no injection	GPS III (Biomet)/ Angel (Arthrex)	LR	1343 ± 670 k/µL 2064 ± 526 k/µL	1	Failure	[52]
Griffin et al.	III	2 arms	15(11)	20(15)	2 years	no injection	Cascade Platelet Rich Fibrin Matrix	N/R	N/R	1	IKDC Tegner Lysholm ROM Failure	[50]
Kemmochi et al.	II	2 arms	17	5	6 months	no injection	N/R	LR 3.6 × NPC (2.0–7.3)	5.5 × NPC (3.4–9.1)	1	Tegner Lysholm IKDC	[49]
Kamiński et al., 2018	I	2 arms	19(18)	18(17)	45 months	saline	N/R	LR	N/R	1	VAS KOOS WOMAC IKDC Failure	[47]
Kamiński et al., 2019	I	2 arms	42(40)	30(29)	54 months	trephination	N/R	LR	823 (320–1659) × 10^3^/µL	1	VAS KOOS IKDC WOMAC Failure	[48]
Pujol et al.	III	2 arms	17(16)	17(15)	2 years	no injection	GPS III (Biomet)	N/R	N/R	1	KOOS IKDC ROM Failure	[51]

LOE—level of evidence; exp.—no. of patients received treatment in experimental group (no. of patients analyzed at final follow-up); cont.—no. of patients receiving treatment in control group (no. of patients analyzed at final follow-up); LR—leukocyte rich; LP—leukocyte poor; ROM—range of movement; PROM—patient related outcome measures; VAS—visual analog scale; WOMAC—Western Ontario and McMaster Universities Osteoarthritis Index; Tegner—Teger Activity Score; Lysholm—Lysholm Knee Scoring Scale; KOOS—Knee injury and Osteoarthritis Outcome Score; Ikeushi—The knee rating scale of Ikeuchi; IKDC—International Knee Documentation Committee.

**Table 8:** Platelet-rich plasma (PRP) compared with control intervention as adjunct treatment for osteoarthritis (blinded RCTs).

	LOE	Type of Study	Exp.	Cont.	Follow-up	Control	Preparation Kit	LR/LP	Platelet Concentration	Number of inj.	PROM	K-L	Ref.
Ahmad et al.	I	2 arms	PRP 45(45)	45(44)	6 months	HA	N/R	LR	N/R	3	VAS IKDC USG	1–3	[66]
Bastos et al. (2018)	II	2 arms	PRP+MSC 9(9)	MSC 9(9)	12 months	MSC	N/R	N/R	10^6^/μL	1	KOOS ROM CFU-F	1–4	[81]
Bastos et al. (2019)	II	3 arms	MSCs 16(15) MSCs + PRP 14(14)	17(16)	12 months	CS	FalconTM	LP	10^6^/µL	1	KOOS ROM	1–4	[74]
Buendia-Lopez et al.	II	3 arms	PRP 35(33)	HA 36(32) NSAIDs 35(33)	52 weeks	HA NSAIDs	N/R	LP	1,095,000 ± 23,200/mm^3^	1	WOMAC VAS X-ray MRI	1–2	[69]
Cole et al.	I	2 arms	PRP 52(49)	59(50)	52 weeks	HA	ACP	LP	1.73 ± 0.053 xNPC	3	WOMAC IKDC VAS Lysholm	1–3	[63]
Duymus et al.	I	3 arms	PRP 39(33)	HA 39(34) Ozone 39(35)	12 months	HA Ozone	Ycellbio kit	LP	>1,500,000/µL	2	WOMAc VAS	2–3	[62]
Elik et al.	I	2 arms	PRP 30(30)	30(27)	6 months	saline	Revmed, VERSUS-5000 i2	LR	N/R	3	VAS WOMAC SF-36 USG	1–3	[57]
Filardo et al. (2012)	II	2 arms	PRP 54	55	12 months	HA	N/R	LR	5 × NPC	3	IKDC EQ-VAS Tegner KOOS ROM	1–3	[78]
Filardo et al. (2015)	I	2 arms	PRP 96(94)	93(89)	12 months	HA	N/R	LR 1.1 ± 0.5 × NLC	4.6 ± 1.4 x NPC	3	IKDC KOOS EQ-VAS Tegner ROM	0–3	[60]
Görmeli et al.	I	4 arms	PRP 3 44(39) PRP 1 45(44)	HA 44(39) Saline 43(40)	6 months	HA saline	N/R	N/R	5.2 × (1,118,000 µL) 5.3 × (1,152,000 µL)	3 1	EQ-VAS IKDC	1–3 or 4	[54]
Jubert et al.	II	2 arms	PRP 35(34)	30(30)	6 months	CS	N/R	LP	0.99 × 10^6^/µL (0.34–1.54 × 10^6^/ µL)	1	VAS KOOS SF-36	3–4	[58]
Kavadar et al.	I	3 arms	PRP1 34(33) PRP2 34(32) PRP3 34(33)	--	6 months	-	N/R	LR	4–5 × NPC	1 2 3	VAS WOMAC TUG	3	[80]
Kon et al.	II	2 arms	APS 31(29)	15(15)	12 months	saline	nSTRIDE APS Kit (Biomet)	LR	N/R	1	VAS WOMAC KOOS SF-36 CGI-S/C PGI-S/C OMERACT –OARSI MRI RTG MRI	2 or 3	[56]
Lana et al.	I	3 arms	PRP 36(36)HA + PRP 33(33)	36(36)	12 months	HA	N/R	LR	800,000–1,600,000/mm^3^	3	WOMAC, VAS	1–3	[79]
Lin et al.	I	3 arm	PRP 31(31)	HA 29(29) S 27(27)	12 months	HA Saline	RegenKit-THT	LP	1.81 ± 0.34 × NPC	3	WOMAC IKDC	Ahlbäck 1–3	[89]
Lisi et al.	I	2 arm	PRP 28(25)	22(22)	12 months	HA	N/R	N/R	N/R	3	WOMAC Lysholm Tegner AKSS Lequesne VAS ROM	2–3	[68]
Louis et al.	II	2 arms	PRP 26(17)	28(17)	6 months	Durolane, HA	MultifugeHeraus R	LP	800 ± 276 × 10^9^/L	1	WOMAC VAS RTG ROM	2–4	[65]
Di Martino et al.	I	2 arms	PRP 96(85)	93(82)	24 months	HA	N/R	1.1 ± 0.5 × NLC	4.6 ±1.4 × NPC	3	IKDC EQVAS Tegner	1–3	[70]
Montañez-Heredia et al.	I	2 arms	PRP 28(27)	27(26)	6 months	HA	N/R	LP	952 × 10^9^/L	1	VAS KOOS EQoL	1–3	[76]
Patel et al.	I	3 arms	PRP1 27(26) PRP2 25(25)	23(23)	6 months	saline	N/R	LP	310.14 × 10^3^/µL	1 2	WOMAC VAS	Ahlbäck 1–2	[86]
Paterson et al.	I	2 arms	PRP 11(10)	10(9)	12 weeks	HA	Premiere XC-2000	LR	N/R	3	VAS KOOS KQoL Functional tests	2–3	[61]
Raeissadat et al. (2017)	II	2 arms	PRGF-Endoret 41(36)	36(33)	6 months	HA	Rooyagen Kit	LR	4.6 ± 0.7 × NPC	2	WOMAC Lequesne VAS	2–3	[71]
Rahimzadeh et al.	I	2 arms	PRP 21(21)	21(21)	6 months	PRL (dextrose)	Standard kit, Iran	N/R	N/R	2	WOMAC	1–2	[73]
Sánchez et al.	I	2 arms	PRGF-Endoret 89(79)	87(74)	6 months	HA	BTI Biotechnology Institute system	LP	N/R	3	WOMAC Lequesne OMERACT–OARSI	Ahlbäck 1–3	[90]
Simental- Mendía et al. (2016)	I	2 arms	PRP 33(33)	32(32)	24 weeks	acetaminophen	N/R	LP	513.25 ± 189.3 K/µL	3	VAS WOMAC, SF-12	1 or 2	[55]
Simental-Mendía (2019)	I	2 arms	1 prp 18 3 prp 17	-	48 weeks	-	NR	LP	99.3 ± 162.0 × 10^6^/μL	1 3	VAS WOMAC SF-12	1–2	[85]
Smith et al.	I	2 arms	ACP 15(15)	15(15)	1 year	saline	Hettich ROTOFIX 32 A; Arthrex	LP	N/R	3	WOMAC	2–3	[72]
Su et al.	I	3 arms	io 28(27) ia 26(25)	32(30)	18 months	HA	N/R	LR 29.92 ± 1.54 × 10^9^/L.	789.68 ± 17.80 × 10^9^/L	2	VAS WOMAC	2–3	[67]
Tavassoli et al.	II	3 arms	PRP 1 31(28) PRP 2 33(28)	31(27)	12 weeks	HA	Rooyagen kit	LR	N/R	1 2	WOMAC VAS	Ahlbäck 1–4	[87]
Uslu-Guvendi et al.	II	3 arms	PRP 1 19(19) PRP3 19(14)	19(17)	6 months	CS	N/R	8.67 10^9^/L	875 10^9^/L	1 3	VNS WOMAC Lequesne	3	[59]
Vaquerizo et al.	I	2 arms	48(48) PRGF-Endoret	48(42)	48 weeks	Durolane HA	BTI Biotechnology Institute system	LP	N/R	3	WOMAC Lequesne OMERACT–OARSI	2–4	[75]
Wu et al.	I	2 arms	20(20)	20(20)	6 months	saline	RegenKit-THT-1, Regen Lab	LR	N/R	1	WOMAC Isokineticfunction	Ahlbäck 1–2	[88]
Yu et al.	II	4 arms	PRP 104 PRP + HA 96	HA 88 saline 72	1 year	HA saline	N/R	N/R	N/R	1	WOMAC Kanofsky	-	[91]

LOE—level of evidence; exp.—no. of patients receiving treatment in experimental group (no. of patients analyzed at final follow-up); cont.—no. of patients receiving treatment in control group (no. of patients analyzed at final follow-up); LR—leukocyte rich; LP—leukocyte poor; ROM—range of movement; PROM—patient related outcome measures; MRI—magnetic resonance imaging; VAS—visual analog scale; WOMAC—Western Ontario and McMaster Universities Osteoarthritis Index; TUG—tug lesion; Tegner—Teger Activity Score; SF-36—36-Item Short Form Survey; SF-12—12-Item Short Form Survey; PGI-S/C—Patient Global Impression of Severity Scale; OMERACT–OARSI—OMERACT–OARSI osteoarthritis pain measure; Lysholm—Lysholm Knee Scoring Scale; Lequesne—Lequesne index of severity for osteoarthritis; KQol—knee-related quality of life; KOOS—Knee injury and Osteoarthritis Outcome Score; K-L—Kellgren–Lawrence scale; Karnofsky—Karnofsky Performance Status Scale; EQ-VAS—EuroQol Visual analogue scale; Eqol—EuroQol quality of life scale; CGI-S/C—The Clinical Global Impressions Scale; CFU—colony forming unit; AKSS—American Knee Society Score.

**Table 9:** Platelet-rich plasma (PRP) compared with control intervention as adjunct treatment for osteoarthritis (non-blinded RCTs).

	LOE	Type of Study	Exp	Cont	Follow-up	Control	Preparation Kit	LR/LP	Plateletconcentration	Number of Inj.	PROM	K-L	Ref
Cerza et al.	I	2 arms	ACP 60(60)	60(60)	24 weeks	HA	ACP (Arthrex)	LP	N/R	4	WOMAC	1–3	[84]
Huang et al.	I	3 arms	40(40)	HA 40(40) CS 40(40)	12 months	HA CS	N/R	LP	N/R	4	WOMAC VAS	1–2	[77]
Raeissadat et al. (2015)	II	2 arms	PRP 87(77)	73(62)	12 months	HA	Rooyagen Kit	LR	4.8 ± 1.80 × NPC	2	WOMAC SF-36	1–4	[82]
Rayegani et al.	I	2 arms	32(31)	33(31)	6 months	acetaminophen	Rooyagen kit	LR	5.6 × NPC	2	WOMAC SF-36	1–4	[64]
Spakova et al.	II	2 arms	PRP 60	60	6 months	HA	Labofuge 400R, Heraeus	LR 6.4 ± 2.3 × 10^3^/µL	680 ± 132 × 10^6^/mL	3	WOMAC NRS-11	1–3	[83]

LOE—level of evidence; exp.—no. of patients receiving treatment in experimental group (no. of patients analyzed at final follow-up); cont.—no. of patients receiving treatment in control group (no. of patients analyzed at final follow-up); LR—leukocyte rich; LP—leukocyte poor; ROM—range of movement; PROM—patient related outcome measures; MRI—magnetic resonance imaging; VAS—visual analog scale; WOMAC—Western Ontario and McMaster Universities Osteoarthritis Index; SF-36—36-Item Short Form Survey; NRS-11—numerical rating scale.
